# RNA *trans*-splicing to rescue β-catenin: A novel approach for treating CTNNB1-Haploinsufficiency disorder

**DOI:** 10.1016/j.omtn.2025.102680

**Published:** 2025-08-12

**Authors:** Matea Maruna, Petra Sušjan-Leite, Maja Meško, Špela Miroševič, Roman Jerala

**Affiliations:** 1 Department of Synthetic Biology and Immunology, National Institute of Chemistry, Hajdrihova 19, 1000 Ljubljana, Slovenia; 2 Graduate School of Biomedicine, University of Ljubljana, Kongresni trg 12, 1000 Ljubljana, Slovenia; 3 Department of Family Medicine, Faculty of Medicine, University of Ljubljana, 1000 Ljubljana, Slovenia; 4 Centre for Technology of Gene and Cell Therapy, National Institute of Chemistry, 1000 Ljubljana, Slovenia

**Keywords:** MT: RNA/DNA Editing, trans-splicing, CTNNB1 syndrome, rare disease, ribozymes, RNA therapy, small antisense RNA

## Abstract

Loss-of-function mutations in the *CTNNB1* gene cause β-catenin deficiency, resulting in CTNNB1 syndrome, a rare neurodevelopmental disorder characterized by motor and cognitive impairments. Given the wide variety of mutations across *CTNNB1* and its dosage sensitivity, a mutation-independent therapeutic approach that preserves endogenous gene regulation is critically needed. This study introduces spliceosome-mediated RNA *trans*-splicing as a novel approach to restore β-catenin production. Pre-*trans*-splicing RNA molecules (PTMs) targeting *CTNNB1* introns 2, 5, and 6 were designed and evaluated using a split yellow fluorescent protein reporter system. Rationally designed short antisense RNAs, which mask splicing regulatory elements, significantly enhanced PTM-mediated *trans*-splicing at both mRNA and protein levels. Additionally, introducing a self-cleaving ribozyme at the PTM’s 5′ end further improved *trans*-splicing efficiency, likely due to increased nuclear retention. CMV promoter-driven PTM expression yielded the highest efficiency. Importantly, successful *trans*-splicing of the endogenous *CTNNB1* transcript confirmed the physiological relevance of this strategy. This study is the first to apply and optimize spliceosome-mediated RNA *trans*-splicing (SMaRT) for *CTNNB1* mRNA correction, providing a promising, mutation-agnostic approach for treating CTNNB1 syndrome.

## Graphical abstract

**Figure:**
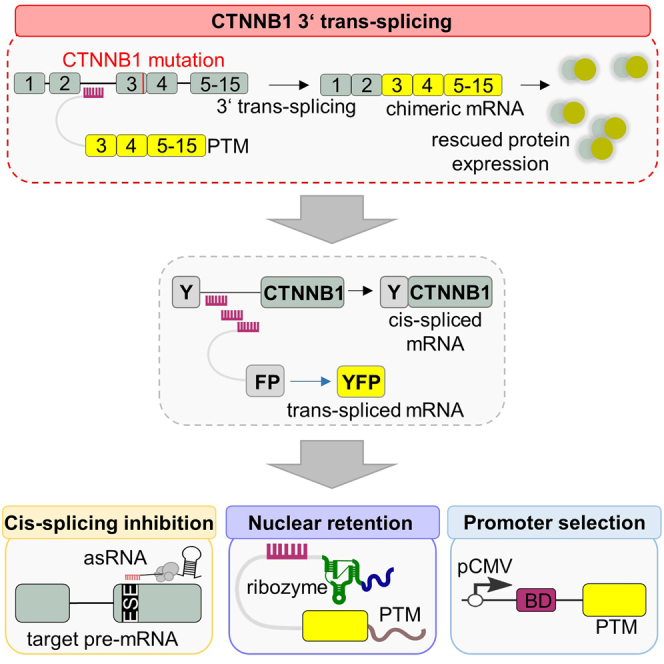

## Teaser

Jerala and colleagues investigate an RNA *trans*-splicing strategy to correct CTNNB1 syndrome mutations and restore β-catenin expression. Using ribozymes, short antisense RNAs, and optimized promoters, they enhance *trans*-splicing in a split YFP reporter system, highlighting its therapeutic potential for CTNNB1 syndrome.

## Introduction

Loss-of-function mutations in the catenin beta 1 (*CTNNB1*) gene lead to β-catenin deficiency, which is strongly associated with the CTNNB1 syndrome, a rare, monogenic, neurodevelopmental disorder marked by an array of motor and cognitive impairments with an estimated prevalence of around 2.6–3.2 in 100,000 live births.^1^ Due to the haplo-insufficiency of the *CTNNB1* gene, the pathological phenotype is driven by a single-allele mutation.^2^^,^^3^^,^^4^ *CTNNB1* mutations vary in type and position and can be found scattered across the whole coding and non-coding region of the *CTNNB1* gene. This advocates the need for a generally applicable approach, capable of correcting a large fraction of diverse mutations, including point mutations and indels.^4^^,^^5^^,^^6^ Furthermore, given that pathogenicity can arise from both β-catenin deficiency due to loss-of-function mutations as well as its overaccumulation due to gain-of-function mutations, the *CTNNB1* gene bears characteristics of a dosage-sensitive gene; therefore, its endogenous gene regulation should preferably be maintained.^7^^,^^8^^,^^9^ Finally, it remains unknown whether the mutated *CTNNB1* transcripts undergo nonsense-mediated RNA decay or translate into truncated variants with potential dominant-negative effects that could hinder the function of the wild-type (WT) β-catenin from the remaining allele. However, the mouse model reportedly exhibits such dominant-negative effect.^10^ Therefore, in some patients, gene upregulation and elimination of the mutated transcript might be necessary to restore the physiological CTNNB1 function.

One of the major processes of mRNA maturation is RNA splicing, in which exons within the nascent mRNA are ligated following intron removal. Splicing is catalyzed by the spliceosome, a large ribonucleoprotein complex, whose formation is orchestrated by five small nuclear RNAs (U1, U2, U4, U5, and U6 snRNA) acting in concert with small nuclear ribonucleoprotein particles (snRNPs) and other protein factors.^11^ The pre-mRNA splicing proceeds in a *cis* manner, with exon ligation occurring within the same pre-mRNA molecule. It could, however, also proceed in a *trans* manner, where exons of different pre-mRNA molecules are combined into a hybrid mRNA in a process known as *trans*-splicing.^11^ Spliceosome-mediated RNA *trans*-splicing (SMaRT) is an artificial exon replacement strategy that utilizes this mechanism, offering potential as a research and therapeutic tool for mRNA repair.^12^

**Figure 1:**
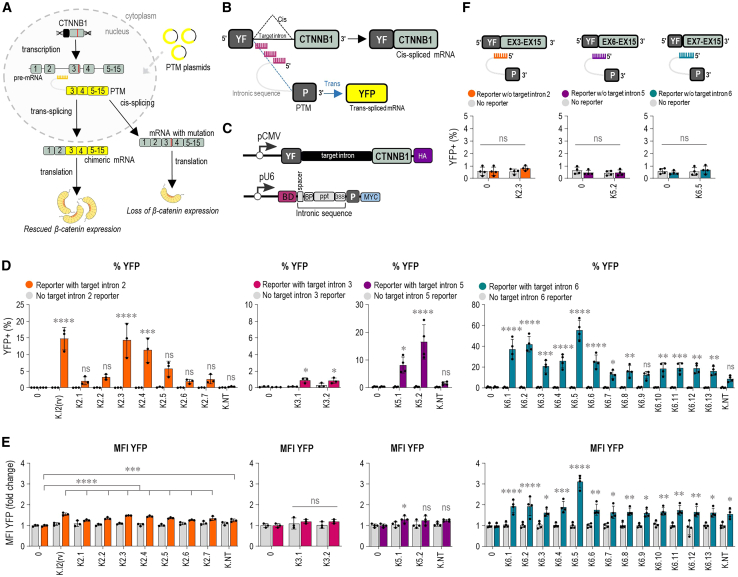
*Trans*-splicing mechanism and PTM candidate screening using a split YFP target intron reporter for efficient *trans*-splicing (A) Following transcription, the *CTNNB1* gene is transcribed into pre-mRNA containing a CTNNB1-associated mutation, leading to the loss of β-catenin expression. A PTM is introduced into the cells to induce *trans*-splicing and outcompete *cis*-splicing, resulting in the formation of chimeric mRNA without the mutation, thereby rescuing β-catenin expression. (B and C) Composition of the split YFP target intron reporter: it consists of the target mRNA and PTM candidate. The target mRNA, expressed under the CMV promoter, contains the N-terminal domain of YFP, a target intron, and downstream exons. The PTM molecule, expressed under the U6 promoter, includes a binding domain (BD), intronic sequence (spacer, branchpoint [BP], polypyrimidine tract [ppt], and 3′ splice site [3′ SS]), the C-terminal domain of YFP, and a myc-tag. Accurate *trans*-splicing results in the fusion of both YFP domains into a functional protein, detected as fluorescence via flow cytometry. (D and E) Quantitation of flow cytometry for PTM screening targeting introns 2, 3, 5, and 6 was performed using the split YFP target intron reporter. Negative controls included PTM K.NT with a randomly generated BD, target intron reporter transfected alone (0), and PTM candidate transfected without the target intron reporter. Quantitation by flow cytometry was presented as the percentage of YFP^+^ cells and the YFP MFI normalized to the target intron reporter-only control (0) for the four different introns—2, 3, 5, and 6. (F) Spontaneous association of the translated N- and C-terminal YFP due to overexpression was tested using flow cytometry. The PTM candidates (K2.3, K5.2, and K6.5) were tested with a reporter lacking the target intron to assess any spontaneous association of the translated N- and C-terminal YFP. The results are presented as the percentage of YFP^+^ cells. The negative controls consisted of the reporter lacking the target intron transfected alone, and PTM transfected alone without the reporter. (D, E, and F) Data are presented as the mean value ±SD from at least three independent experiments. Comparison to the target intron reporter only (target only) was analyzed using ordinary one-way ANOVA with Dunnett’s multiple comparison test. ∗∗∗∗*p* < 0.0001; ∗∗∗*p* < 0.001; ∗∗*p* < 0.01; ∗*p* < 0.05; nonsignificant (ns).

It is based on the rational design of an exogenous pre-*trans*-splicing RNA molecule (PTM) capable of replacing selected exons in a target pre-mRNA molecule, thereby generating a chimeric mRNA (Figure 1A). Depending on the region to be replaced in the target pre-mRNA, there are three types of *trans*-splicing: 3′, 5′, and internal *trans*-splicing.^13^ SMaRT strategy allows for a partial gene replacement, which is particularly beneficial in the correction of large genes unsuitable for introduction through adeno-associated virus (AAV) vectors, correction of genetic defects regardless of their size and type, and, most importantly, it maintains the endogenous transcriptional regulation of the target gene in terms of time, space, and transcriptional output. To date, *trans*-splicing has been demonstrated in several cell models of dominant and recessive genetic diseases such as cystic fibrosis,^14^^,^^15^ spinal muscular atrophy,^16^ Duchenne muscular dystrophy,^17^^,^^18^ dystrophic epidermolysis bullosa,^19^^,^^20^ and retinitis pigmentosa.^21^^,^^22^ Furthermore, *trans*-splicing has been recently reported as an elegant tool to facilitate the reconstitution of a split gene following dual AAV vector delivery.^23^ While these successes highlight *trans*-splicing as a promising therapeutic strategy, its dissemination, both clinically and as a biochemical research tool, has been significantly hindered by its low efficiency.^24^ Optimization of the original PTM design, such as the selection of a suitable target intron and binding domain (BD), the position and length of the BD, the presence of mismatch mutations, the thermodynamic stability of PTM terminal ends, and most recently, implementations of the CRISPR-Cas13 system, can significantly improve *trans*-splicing efficiency.^25^^,^^26^^,^^27^^,^^28^^,^^29^

This study is the first to investigate the feasibility of the SMaRT strategy for the replacement of CTNNB1 exons. Several *CTNNB1* characteristics highlight *trans*-splicing as a promising avenue to pursue in search of treatment for the CTNNB1 syndrome. The above-mentioned issues regarding *CTNNB1* could be elegantly avoided with a *trans*-splicing-based intervention, as it would eliminate the effect of diverse mutations and restore functionality through the exon replacement while retaining the endogenous gene regulation. We introduced both the initially reported PTM design and additional modifications aimed to enhance SMaRT efficiency. Upon PTM screening within a split yellow fluorescent protein (YFP) reporter system, we identified the best PTM candidates for three target introns within the *CTNNB1* gene, with *trans*-splicing efficiency ranging from 15% to 55%. *Trans*-splicing efficiency was strongly enhanced by the addition of the rationally designed short antisense RNAs (asRNAs), expressed in a hybrid U1-U7 snRNA cassette that targets splicing regulatory elements and inhibits *cis*-splicing. Furthermore, the choice of the CMV promoter for the PTM expression and novel modification of positioning a self-cleaving ribozyme on its 5′ significantly improved PTM-mediated *trans*-splicing targeting introns 2 and 5. This improvement allowed us to achieve the *trans*-splicing efficiency of the Cas13 by implementing *trans*-splicing techniques in the reporter system. For these introns, we were able to detect robust *trans*-splicing of the endogenous *CTNNB1* transcript, indicating the physiological and potentially therapeutic relevance of this strategy.

## Results

### Design of the PTM candidates supporting *trans*-splicing of *CTNNB1* introns

In this study, we aimed to optimize 3′ *trans*-splicing for the *CTNNB1* gene that could correct most reported mutations that cause CTNNB1 syndrome (Figure 1A) while maintaining the endogenous transcriptional regulation. As the introns with stronger splice sites are more likely to favor *cis*-splicing and outcompete PTM,^24^ we first identified the most suitable *CTNNB1* intron candidates for *trans*-splicing based on the computational prediction of their splice site (SS) strength using the MaxEnt.^30^ We selected for experimental testing *CTNNB1* introns 2, 3, 5, and 6 as *trans*-splicing targets as they exhibited low MaxEnt scores, while a strong 3′ SS was introduced in the designed PTM candidates (Table S1). Furthermore, targeting intron 2 would allow replacement of most of the coding region of the *CTNNB1* transcript, making this approach widely applicable to CTNNB1 syndrome-associated mutations, which are spread across the entire gene. Similarly, the effective targeting of introns 6 and 5 could be applied for many of the reported mutations, as most mutations are located in exons 7 and 8.^4^ Previous studies have shown that the selection of suitable BD within an intron preceding the exons to be replaced is crucial in the design of an efficient PTM.^29^^,^^31^ To this end, we utilized a tiling screening in which we designed PTM candidates with 150-nucleotide (nt) long BDs spanning the entire sequence of target introns 2, 3, and 6 with 50-nt overlaps. In terms of targeting intron 2, we also designed a BD as a reverse complement of the entire intron 2. As intron 5 is shorter, the BD length for intron 5 screening was 50-nt, with a 15-nt overlap. Eight PTM molecules differing in their BD, complementary to different sites within the target intron, were screened for intron 2, 14 for intron 6, and two PTMs for introns 3 and 5 (Figures S1 and S2).

To screen for PTM candidates with the highest *trans*-splicing efficiency, we designed a split YFP reporter system, which consisted of the construct comprising the target *CTNNB1* intron (hence target intron reporter) and PTM in fusion with N- and C-terminal parts of the *YFP* gene, respectively (Figure 1B). Target intron reporter comprised the N-terminal domain of the *YFP* gene, target intron, downstream exons from the *CTNNB1* gene, and the hemagglutinin (HA) tag (Figure 1C). PTM candidates were designed for 3′ *trans*-splicing and comprised BD, spacer, an intronic sequence including branchpoint (BP), polypyrimidine tract (ppt), and 3' acceptor SS, followed by the C-terminal domain of the *YFP* gene and myc-tag (Figure 1C). In this reporter system, *trans*-splicing between the target intron reporter and PTM molecule in cells is expected to assemble a functional YFP whose fluorescence can be measured by flow cytometry (Figure 1B).

On co-transfection of the target intron reporter and corresponding PTM into HEK293T cells at a molar ratio of 1:2, flow cytometry was used to measure the percentage of cells expressing YFP and median fluorescence intensity (MFI) of the YFP fluorescence. Specifically, cells were gated for the 20,000 iRFP^+^ cells, which served as a control for transfection efficiency, and were further analyzed for YFP expression, indicating the success of *trans*-splicing (Figure S3). An increase in the percentage of YFP^+^ cells from 15% to over 40% was detected with the best PTM candidates (K2.3, K6.5, and K5.2) in comparison with the control (Figure 1D). Accordingly, we detected an increase in the YFP MFI, especially in the case of intron 6, where a 3-fold increase was detected (Figure 1E). Neither the target intron reporter nor PTM showed any increase in YFP fluorescence when transfected alone (Figures 1D and 1E). Importantly, the signal was associated with the presence of the BD with base complementarity to the target intron as a PTM K.NT with random RNA sequence in place of the BD, resulting in little to no increase in the YFP signal compared with the tested PTMs (Figures 1D and 1E). Furthermore, we also performed co-transfection of the best PTM candidates (K2.3, K6.5, and K5.2) with a reporter lacking the target intron to control for any spontaneous association of the translated N- and C-terminal YFP as a result of the overexpression. No increase in YFP signal was observed, indicating the absence of interaction between translated N- and C-terminal YFP in HEK293T cells (Figure 1F). PTM candidates targeting intron 3 did not lead to an increase in the percentage of YFP^+^ cells or YFP MFI and were excluded from further experiments (Figures 1D and 1E).

We investigated the effects of the length of the BD. Initially, we designed 150-nt-long BDs, which has been reported before to provide the best *trans*-splicing efficiency, and least off-target effects in comparison with shorter or longer BDs.^29^ Stability of shorter RNA duplexes, such as 100 or even 50-nt, is expected to be similar to the 150 nt. Therefore, to assess the impact of shorter BD, we shortened the BDs of best-performing PTM candidates, K2.3 and K6.5, to 100-nt and 50-nt. We tested truncations from the 5′, 3′, and both ends of the BD. All BD truncations led to a significant decrease in the *trans*-splicing efficiency in all tested PTM candidates (Figures S4A and S4B). An explanation could be that longer BDs may be able to disrupt stable secondary structures within the mRNA through strand displacement as they have a higher probability to anneal to the single-stranded regions within the mRNA than shorter segments.

### Antisense RNA strongly enhances PTM-mediated *trans*-splicing

**Figure 2:**
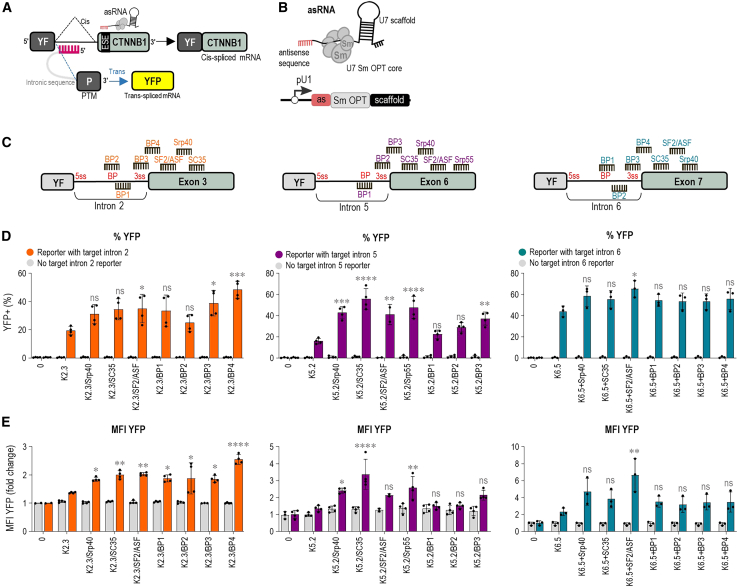
Short asRNAs enhance *trans*-splicing efficiency for *CTNNB1* introns in the target intron reporter (A) A schematic representation illustrates the mechanism by which short asRNAs block *cis*-splicing. The 30- to 35-nt-long asRNAs are designed to target regions from the BP to the 3′ SS in the target intron, as well as ESEs within the first exon downstream of the target intron. (B) The short asRNAs consist of an antisense sequence (as) coupled with a modified U7 Sm OPT core, which binds Sm proteins found in spliceosomal snRNAs, along with a U7 snRNA scaffold. Chimeric U7-antisense sequences are expressed under the control of a strong Pol II U1 snRNA gene promoter and termination sequence. (C) The positions of short asRNAs targeting SSs and ESEs are mapped in target introns 2, 5, and 6, and their respective first downstream exons 3, 6, and 7. (D and E) The detection of enhanced *trans*-splicing efficiency by adding short asRNAs was performed using flow cytometry. Negative controls included cells transfected with the target intron reporter alone (0), the PTM candidate transfected alone, or the PTM candidate and asRNA transfected alone. Quantitation by flow cytometry is presented as the percentage of YFP^+^ cells (D) and the YFP MFI (E) normalized to the reporter-only control (0) for the three different introns—2, 5, and 6. Data are presented as the mean value ±SD from at least three independent experiments. Comparison to the PTM candidate co-transfected with the target intron reporter was analyzed using ordinary one-way ANOVA with Dunnett’s multiple comparison test. ∗∗∗∗*p* < 0.0001; ∗∗∗*p* < 0.001; ∗∗*p* < 0.01; ∗*p* < 0.05; nonsignificant (ns).

We next aimed to improve the *trans*-splicing efficiency by introducing various modifications to the original PTM design to either inhibit the *cis*-splicing, enhance its nuclear retention, prevent PTM degradation, or prevent translation of PTM in the absence of splicing. Combination of PTM with random antisense sequences and synthetic antisense oligonucleotides (ASOs) targeting specific intron-exon junctions was reported to boost *trans*-splicing, presumably by inhibiting the competitive *cis*-splicing.^20^ To suppress *cis*-splicing, we opted for an addition of short asRNA with complementarity to the segments that contain BP, 3′ SS, and exon splicing enhancers (ESE) within the target intron and the first downstream exon (Figure 2A). To support robust asRNA expression combined with enhanced nuclear retention and resistance to degradation, asRNAs were expressed in a hybrid U1-U7 expression cassette in which the 5′ end of the modified U7 snRNA was replaced by the antisense sequence of choice expressed under a strong, DNA polymerase II-dependent snRNA U1 promoter^32^^,^^33^ (Figure 2B). We designed 30-nt-long asRNAs masking BP and 3′ SS in the target intron-exon junction and 30- to 35-nt-long asRNAs masking selected ESE within the first downstream exon (Figure 2C). Designed asRNAs were co-transfected into HEK293T cells in combination with the best corresponding PTM from screening in Figures 1D and 1E and the target intron reporter. Flow cytometry revealed that the addition of short asRNAs significantly enhanced *trans*-splicing efficiency of PTM candidates for all target introns, as observed both in percentage of YFP^+^ cells and YFP MFI (Figures 2D and 2E). The asRNA candidate BP4 in the case of intron 2 *trans*-splicing increased the percentage of YFP^+^ cells from 19% to 48%, while the best asRNA SF2/ASF for intron 6 *trans*-splicing improved the YFP^+^ cell percentage from 44% to 65%. In case of intron 5 *trans*-splicing, asRNA SC35 targeting exon 6 increased the YFP^+^ cells from 16% to 55% (Figure 2D). Improved YFP production was also observed in terms of YFP MFI, with the highest increase detected for intron 6 (Figure 2E).

We next wanted to see if the expression of multiple asRNAs would further enhance PTM-mediated *trans*-splicing in target introns 2 and 6. However, a combination of two or three asRNAs, expressed from a single vector, led to no further improvement in *trans*-splicing efficiency targeting introns 2 and 6 in the split YFP reporter system (Figures S4C and S4D).

Since both the U6 and U1 promoters used in this study are commonly employed for non-coding RNA expression,^34^^,^^35^^,^^36^ we investigated whether combining PTMs K2.3 and K6.5 with antisense sequences from asRNAs, all expressed under the U6 promoter in a single vector, produces the same effect as when PTMs and asRNAs are expressed under the separate promoters. This single-vector strategy aimed to reduce the complexity of the system, ensuring the co-expression of both the asRNA and PTM within the same cell, and potentially improve the coordination of their activity on the target pre-mRNA. For the combined PTMs K2.3 and K6.5, we inserted the antisense sequence from the best-performing asRNAs upstream of the BD. We designed the combined PTMs with a 3-nt spacer between the asRNA sequence and the BD to increase flexibility and allow both the BD and the antisense sequence to effectively bind to their respective target sites. However, flow cytometry results indicated that the combined PTMs K2.3 and K6.5 with asRNA in a single vector were less efficient than when PTMs and asRNAs were expressed under separate promoters and vectors. This was especially evident from the significant decrease in YFP MFI for both introns (Figures S4E and S4F). The reduced efficiency suggests that a single chain may constrain binding to the two sites at the same time, which might be solved by either a longer linker or self-cleaving RNA.

A combination of asRNA and PTM resulted in an increased *trans*-splicing efficiency that was consistently substantially enhanced compared with PTM alone (Figures 2D and 2E). This observation, combined with the spatial proximity between the binding site of asRNA and the binding site of PTM on the target pre-mRNA molecule, prompted us to investigate whether asRNA could serve as a replacement for BD of the PTM. We therefore attempted to create a single, hybrid *trans*-splicing molecule in which we attached all of the PTM constituents except for the BD (spacer, BP, ppt, 3′ SS, C-YFP, and myc-tag) to the 5′ of the respective asRNA in a U1-U7 expression cassette (Figure S5A) and tested whether it could convey the same *trans*-splicing efficiency as the combination of separate asRNA and PTM molecules. While such a construct supported *trans*-splicing, it was less efficient than the combination of PTM and asRNA from two separate constructs as well as the original PTM alone (Figure S5B), which indicates that despite yielding low *trans*-splicing efficiency on its own, a PTM with its own BD is indispensable for efficient *trans*-splicing in our system.

**Figure 3:**
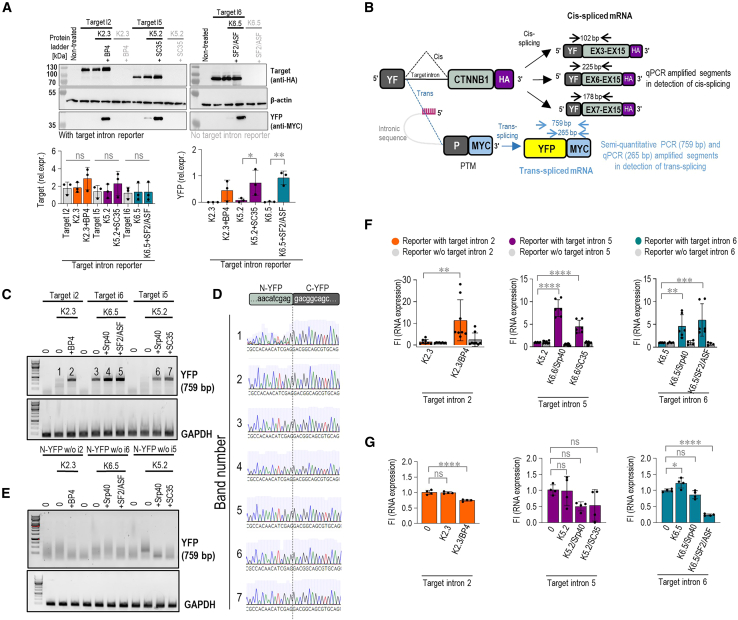
Detection of *trans*-splicing efficiency at the mRNA and protein levels (A) Western blot depicting reconstituted YFP upon PTM and asRNA-facilitated *trans*-splicing of the reporters containing target introns 2, 5, and 6. On transfection, cell lysates were subjected to SDS-PAGE and western blotting with *cis*-spliced reporter detected via HA-tag and *trans*-spliced reporter detected via nYFP-myc-tag. A representative western blot of three independent experiments is shown. 0 represents negative control–cells transfected with an empty plasmid vector (pcDNA3); T represents negative control–target intron reporter transfected alone; PTM and asRNA transfected with target intron reporter are marked in black; PTM and asRNA transfected without target intron reporter are marked in gray. All uncropped blots used for analysis can be found in Figure S12. (B) A schematic representation illustrating the position and size of qPCR and semi-qPCR amplified segments in the detection of *cis*- and *trans*-splicing in (C–G). (C–E) Gel electrophoresis representing semi-qPCR-amplified segments of nYFP-myc-tag junction (759 bp), indicative of reporter *trans*-splicing. Bands numbered 1–7 (C) were isolated from the gel and submitted to Sanger sequencing, with the obtained sequences corresponding to the nYFP-myc-tag junction (D). (F) qPCR-determined relative abundances of the mRNA segment from the cYFP-myc-tag junction (265 bp) indicative of *trans*-splicing. Fold increase is calculated compared with samples transfected with PTM only. GAPDH was used as the reference, and the ΔΔCT method was used for quantification. (G) qPCR-determined relative abundances of the segment from the nYFP and *cis*-spliced exon junction indicative of *cis*-splicing. Fold increase is calculated compared with samples transfected with PTM only. GAPDH was used as the reference, and the ΔΔCT method was used for quantification. (A) Statistical comparisons to the reporter-only control (0) and among tested groups were performed using ordinary one-way ANOVA followed by Dunnett’s multiple comparisons test. ∗∗*p* < 0.01; ∗*p* < 0.05; nonsignificant (ns). (F–G) Comparison of PTM candidate and asRNA co-transfection to PTM candidate only was analyzed using two-way (F) or ordinary one-way (G) ANOVA with Dunnett’s multiple comparison test. ∗∗∗∗*p* < 0.0001; ∗∗∗*p* < 0.001; ∗∗*p* < 0.01; ∗*p* < 0.05; nonsignificant (ns).

Results obtained by flow cytometry were further confirmed by confocal microscopy, where the strongest YFP signal was detected with the combination of PTM and asRNA (Figure S6). Western blot analysis using anti-myc antibodies revealed the presence of full-length YFP bands in cell lysates. When PTMs K5.2 and K6.5 were transfected with the target intron reporter, a YFP band was observed. The addition of asRNAs further significantly increased the YFP signal, indicating enhanced *trans*-splicing efficiency. In contrast, no YFP signal was detectable when PTM K2.3 was transfected with the target intron reporter. However, co-transfection with asRNAs led to the appearance of a strong YFP signal (Figure 3A). In addition, quantitation of the western blot analysis showed no significant differences in target intron reporter protein expression (Figure 3A). *Trans*-splicing was also confirmed on a transcriptional level by semi-quantitative PCR (semi-qPCR). On co-transfection of HEK293T with the target intron reporter, corresponding best PTM candidates (K2.3, K5.2, or K6.5), and asRNA (BP4, SRP40, SF2/ASF, SC35), RNA was isolated from the cell lysates and reverse transcribed to cDNA. *Trans*-splicing was confirmed through amplification of a 759-base pair (bp)-long segment by primers annealing to N-YFP and myc-tag, which are expected to co-localize in the *trans*-spliced reporter molecule (Figure 3B). The bands obtained on agarose gel electrophoresis corresponded to the expected size of the segment (Figure 3C), and Sanger sequencing of the segments extracted from the gel additionally confirmed that *trans*-splicing-mediated joining of N-YFP and C-YFP segments took place at the mRNA level (Figure 3D). No bands were observed in the case of reporters lacking the target introns (Figure 3E). Next, a 265-bp segment of the N-YFP-C-YFP junction was amplified by qPCR to quantify the *trans*-splicing efficiency increase by asRNA (Figure 3B). The values were normalized to the expression of GAPDH and the *trans*-splicing with PTM alone. Following the flow cytometry measurements, *trans*-splicing was strongly enhanced when PTM was combined with the asRNA. Specifically, a 10-fold increase in the YFP junction expression was noted with a combination of PTM K2.3 and BP4 compared with K2.3 alone. PTM 6.5 and PTM K5.2 in combination with the respective asRNA led to a 5- to 10-fold increase in the YFP junction expression (Figure 3F). This increase was dependent on the presence of the target intron-bearing reporter (Figure 3F). A significant decrease in *cis*-splicing of the target intron reporter was detected for the combination of PTM K2.3 and K6.5 with asRNAs when we amplified a segment between N-YFP and the downstream *CTNNB1* exon, which is expected to remain co-localized in a *cis*-spliced target intron reporter (Figure 3G).

### Self-cleaving ribozymes at 5′ of the PTM significantly enhance 3′ *trans*-splicing

**Figure 4:**
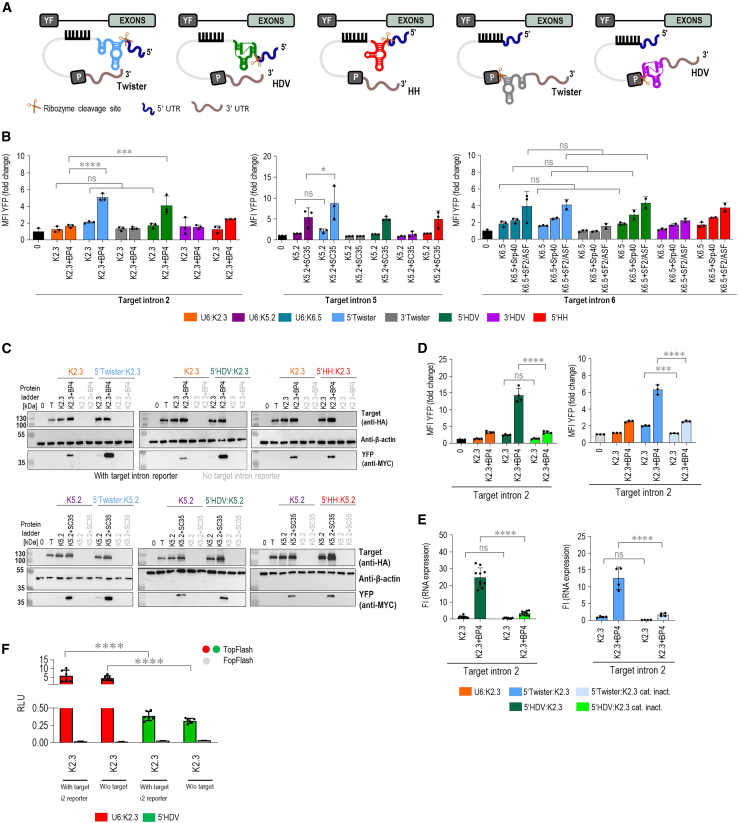
Addition of Twister and HDV ribozymes to the 5′ end of PTM enhanced *trans*-splicing efficiency (A) Schematic example of a PTM targeting intron with added ribozymes on either 5′ or 3′ ends to remove capping or polyadenylation. (B) Determination of *trans*-splicing efficiency for the PTM K2.3, PTM K5.2, and PTM K6.5 by using flow cytometry. Negative control included the target intron reporter transfected alone (0). Quantitation by flow cytometry is presented as the YFP MFI normalized to the reporter-only control (0) for the introns 2, 5, and 6. Data are presented as the mean value ±SD from at least three independent experiments. (C) Detection of *trans*-splicing efficiency for the PTM K2.3 and K5.2 with ribozymes placed at the 5′ end as restoration of full-length YFP by western blot analysis using the corresponding anti-myc, anti-HA, and β-actin antibodies. 0 represents negative control–cells transfected with an empty plasmid vector (pcDNA3); T represents negative control–target intron reporter transfected alone; PTM and asRNA transfected with target intron reporter are marked in black; PTM and asRNA transfected without target intron reporter are marked in gray. Data are representative of three independent experiments. All uncropped blots used for analysis can be found in Figures S13 and S14. (D and E) A decrease in *trans*-splicing efficiency of PTM K2.3 bearing catalytically inactive HDV and Twister ribozyme on its 5′ end was measured by flow cytometry measurement of reconstituted YFP (D) or via qPCR-determined relative abundance of nYFP-myc-tag junction (E). Negative control (0) for the flow cytometry included the target intron reporter transfected alone. Results are presented as the YFP MFI normalized to the reporter-only control (0). qPCR results are expressed as fold increase, calculated compared with samples transfected with PTM only. GAPDH was used as the reference, and the ΔΔCT method was used for quantification. (F) Relative luminescence upon co-transfection of TopFlash reporter plasmid with optimized K2.3 and/or target intron reporter with or without target intron. (B–F) Data are presented as the mean value ±SD from at least three independent experiments. (B, D, and F) Comparison between the tested groups was analyzed using one-way ANOVA with Dunnett’s multiple comparison test. ∗∗∗∗*p* < 0.0001; ∗∗∗*p* < 0.001; ∗*p* < 0.05; nonsignificant (ns).

Next, we explored self-cleaving ribozymes as a tool to cleave off 5′ or 3′ ends of the PTM as a means of manipulating posttranscriptional processing, such as polyadenylation and capping, that importantly affect RNA trafficking from the nucleus, its stability, and translation.^37^^,^^38^ Ribozymes placed at the 3′ of the PTM have been shown to increase 5′ *trans*-splicing efficiency through cleavage of the polyadenylation tail.^28^ The polyadenylation tail of the PTMs designed for 5′ *trans*-splicing is not *trans*-spliced into the target mRNA and therefore only facilitates the undesirable nuclear export and translation of the PTM while being redundant for *trans*-spliced mRNA. We applied the analogous logic to our PTMs designed for 3′ *trans*-splicing and assumed an equally redundant role of 5′ positioned cap. In this, to our knowledge, the first instance of this strategy, we positioned various ribozymes at the 5′ end of the selected PTM candidates for the introns 2, 5, and 6 (K2.3, K5.2, and K6.5). We tested the effect of three self-cleaving ribozymes (Twister, the genomic version of hepatitis D virus [HDV], and hammerhead [HH]) positioned either at 5′ or at 3′ of the three best PTM candidates K2.3, K5.2, and K6.5 (Figure 4A). We observed an increase in the YFP signal with 5′ placement of the HDV and Twister ribozymes on K2.3, which was further significantly increased by the addition of asRNA BP4 (Figure 4B). Furthermore, the placement of the Twister ribozyme at the 5′ end of K5.2 significantly enhanced *trans*-splicing efficiency, particularly when combined with the asRNA SC35 (Figure 4B). The ribozymes did not appear to additionally increase the *trans*-splicing efficiency of K6.5 (Figure 4B). These findings were corroborated by qPCR (Figure S7A) and western blot analysis (Figure 4C). Western blot analysis confirmed a significant increase in *trans*-splicing efficiency when PTM K2.3 with either 5′Twister or 5′ HDV ribozymes was combined with asRNA BP4 (Figures 4C and S7B). In contrast to flow cytometry results, western blot analysis showed a significant increase when K5.2 with 5′ HDV was combined with asRNA SC35 (Figures 4C and S7C). Interestingly, we also observed a significant increase in expression of the *cis*-spliced target intron reporter following co-transfection with PTM K2.3 containing 5′ HDV and PTM K5.2 containing 5′ HH (Figures 4C, S7B, and S7C). This enhancement may result from the stabilization of the target intron reporter on PTM binding or from the overexpression of the constructs in the transfected cells. As a control, we observed a trend toward increased *trans*-splicing efficiency at the RNA level with 3′ placement of ribozymes, which are expected to cleave the polyadenylation tail of the PTM (Figure S7A), likely due to enhanced retention of the PTM within the nucleus. However, no corresponding increase in YFP signal was observed at the protein level with either of the target intron reporters (Figure 4B). This was expected due to the impaired translation of the *trans*-spliced mRNA, lacking the polyadenylation tail. Overall, these findings suggest that the truncation of the 5′ of PTM, which likely lacks capping, is responsible for the increased efficiency.

To confirm that the improvement in *trans*-splicing efficiency observed after positioning the ribozyme to the 5′ end of the PTM molecule is indeed due to the ribozyme’s catalytic activity and not due to stabilization by the formation of a secondary RNA structure, we selected PTM K2.3 targeting intron 2 and design PTM K2.3 containing 5′ HDV and 5′Twister ribozymes with mutations in their respective critical bases: C75 > A (catalytical site of HDV)^39^ and G7 (important for Twister folding).^40^ Flow cytometry analysis revealed that PTM K2.3 with the mutated 5′ HDV and 5′Twister ribozymes exhibited reduced *trans*-splicing efficiency, especially in the presence of asRNA, where a significant decrease in both YFP^+^ cells and MFI was observed. The efficiency of PTM K2.3 containing the mutated ribozymes was comparable to that of PTM K2.3 without any prior optimization (Figures 4D and S7D). These results were further validated by qPCR, which showed the same trend in *trans*-splicing efficiency in the presence of the catalytically inactive ribozymes (Figure 4E). Additionally, when we replaced C-YFP in our selected intron 2 - targeting PTM with the *CTNNB1* exons (3–15), we observed that the original unoptimized PTM K2.3 under U6 promoter exhibited significant translation into active β-catenin, which was detectable by the TopFlash reporter assay (Figure 4F). Partially active β-catenin translation from this PTM is possible because it contains most of the coding *CTNNB1* exons (3–15). In line with our findings, the inclusion of both HDV and Twister ribozyme significantly reduced translation of PTM (Figure 4F) into active β-catenin, which again indicates that ribozyme-mediated de-capping prevents leakage of PTM into the cytoplasm and keeps it within the nucleus.

### Expression under the CMV promoter enhances *trans*-splicing at introns 2 and 5, targeting PTM

**Figure 5:**
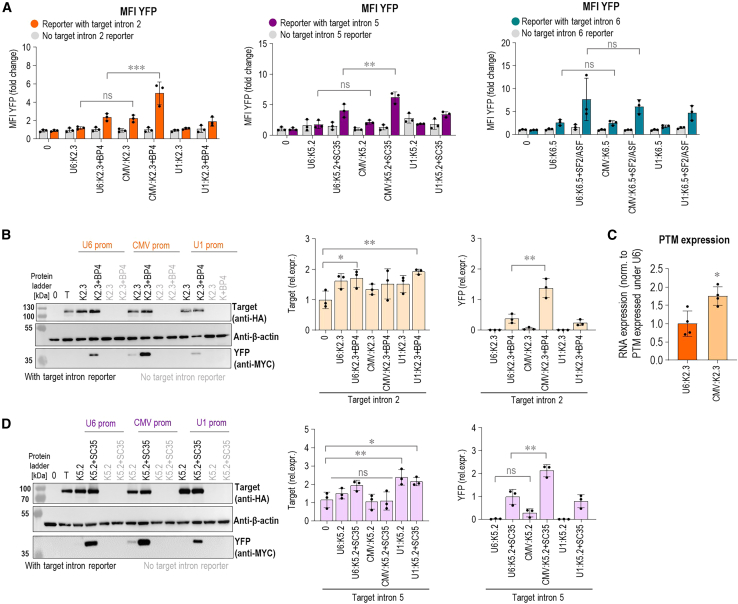
Expression of PTMs K2.3 and K5.2 under the CMV promoter improved *trans*-splicing efficiency (A) Testing of PTM K2.3, K5.2, and K6.5 expressed under U6, CMV, and U1 promoters for the *trans*-splicing efficiency using flow cytometry. Negative controls included cells transfected with the target intron reporter alone, the PTM candidate alone, the PTM candidate, and asRNA without the target intron reporter. Results are shown as YFP MFI normalized to negative control–target intron reporter transfected alone (0). Data are presented as the mean value ±SD from at least three independent experiments. Comparison between the tested groups was analyzed using one-way ANOVA with Dunnett’s multiple comparison test. ∗∗∗∗*p* < 0.0001; ∗∗*p* ≤ 0.01; ∗*p* < 0.05; nonsignificant (ns). (B and D) *Trans*-splicing efficiency of PTM K2.3 (B) and PTM K5.2 (D) expressed under U6, CMV, and U1 was verified using western blot. 0 represents negative control–cells transfected with an empty plasmid vector (pcDNA3); T represents negative control–target intron reporter transfected alone; PTMs and asRNAs transfected with target intron reporter are marked in black; PTMs and asRNAs transfected without target intron reporter are marked in gray. Data are representative of three independent experiments. Analysis of three biological replicates is provided in the charts and presented as the mean value ±SD. Myc-tag (YFP) levels were first normalized to β-actin and then to HA-tag (target). HA-tag levels were normalized to β-actin. Samples with no detectable band signal were quantified as zero. Comparison between the tested groups was analyzed using one-way ANOVA with Dunnett’s multiple comparison test. ∗∗*p* ≤ 0.01; ∗*p* < 0.05; nonsignificant (ns). All uncropped blots used for analysis can be found in Figure S15. (C) Comparison of PTM K2.3 expressed under U6 and CMV promoters by using qPCR with specific primers designed to detect PTM expression. Expression of K2.3 from CMV was normalized to expression of K2.3 expressed under the U6 promoter. Comparison to K2.3 expressed under the U6 promoter was analyzed using Student’s t test (two populations). ∗*p* < 0.05; nonsignificant (ns).

PTMs in the above-described experiments were expressed under the snRNA U6 promoter to support RNA stability and nuclear retention characteristics for non-coding RNAs.^35^ To further improve *trans*-splicing efficiency, we compared the expression of the best-performing PTM candidates under U1, U6, and CMV in the split YFP reporter system. Notably, K2.3 expressed under the CMV promoter exhibited a significant increase in *trans*-splicing efficiency, as evidenced by the percentage of YFP^+^ cells, compared with the PTM expressed under the U6 promoter (Figure S8A). The addition of asRNA BP4 further significantly improved *trans*-splicing efficiency, as observed by the YFP MFI (Figure 5A). Since we observed a significant increase in *trans*-splicing efficiency only for PTM K2.3 expressed under the CMV promoter, we hypothesized that this improvement is due to higher PTM expression levels driven by the CMV promoter compared with the U6 promoter. This was confirmed by qPCR with primers designed to detect PTM expression, where we indeed detected higher expression of PTM K2.3 when expressed under the CMV promoter (Figure 5C). A significant increase in YFP signal was also observed for the best-performing PTM, K5.2, expressed under the CMV promoter in combination with asRNA SC35 (Figures 5A and S8B). In contrast, the CMV promoter did not significantly improve *trans*-splicing by K6.5 PTM (Figures 5A and S8C). Improved *trans*-splicing by K2.3 and K5.2 PTMs expressed under the CMV promoter was further validated by western blot analysis. Western blot analysis showed a significant increase in YFP signal when both PTMs K2.3 and K5.2 were combined with asRNAs (Figures 5B and 5D). We also observed a significant increased expression of *cis*-spliced target intron reporter when co-transfected with PTMs K2.3 and K5.2, both expressed under the U1 promoter.

**Figure 6:**
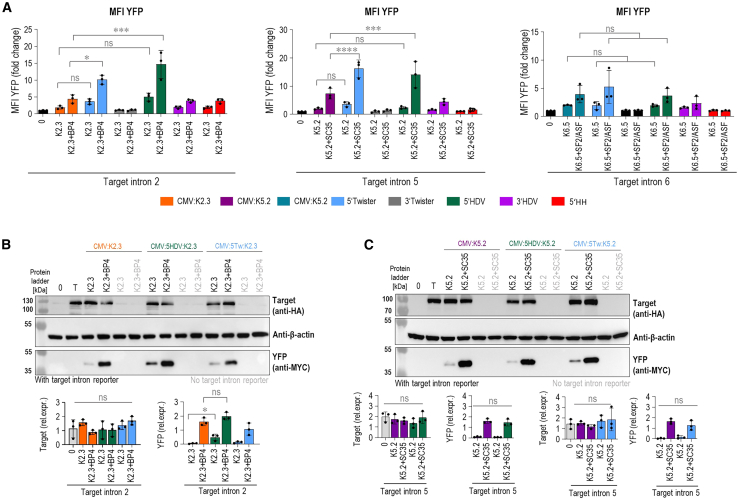
Combination of 5′ ribozymes and CMV expression of PTM K2.3, K5.2, and K6.5 leads further to increased *trans*-splicing efficiency (A) Testing of PTM K2.3, K5.2, and K6.5 expressed under the CMV promoter with added HH, HDV, and Twister ribozymes on either 5′ or 3′ end of PTM. *Trans*-splicing efficiency is detected as reconstitution of YFP detected with flow cytometry. Negative control included the target intron reporter transfected alone (0). Results are presented as the YFP MFI normalized to the reporter-only control. Data are presented as the mean value ±SD from at least three independent experiments. (B and C) Western blot analysis of *trans*-splicing efficiency induced by PTMs K2.3 and K5.2 expressed under the CMV promoter with either 5′ HDV or Twister ribozyme. 0 represents negative control–cells transfected with an empty plasmid vector (pcDNA3); T represents negative control–target intron reporter transfected alone; PTMs and asRNAs transfected with target intron reporter are marked in black; PTMs and asRNAs transfected without target intron reporter are marked in gray. Data are representative of three independent experiments. Analysis of three biological replicates is provided in the charts and presented as the mean value ±SD. Samples with no detectable band signal were quantified as zero. Myc-tag (YFP) levels were first normalized to actin and then to HA-tag (target). HA-tag levels were normalized to actin. All uncropped blots used for analysis can be found in Figure S16. (A–C) Data are representative of three independent experiments. Comparison between the tested groups was analyzed using one-way ANOVA with Dunnett’s multiple comparison test. ∗∗∗∗*p* < 0.0001; ∗∗∗*p* < 0.001; ∗*p* < 0.05; nonsignificant (ns).

After this optimization, we further examined the ability of PTM candidates to induce *trans*-splicing, combining all enhancements that improved *trans*-splicing efficiency. The best-performing PTM candidates K2.3, K5.2, and K6.5 were expressed under the CMV promoter, with self-cleaving ribozymes HH, HDV, and Twister added to either the 5′ or 3′. The optimized PTM K2.3 showed a further increase in *trans*-splicing efficiency on the target intron reporter when the HDV or Twister ribozymes were attached to the 5′ (Figures 6A and S9A). Notably, combining PTM K2.3 with asRNA BP4 led to a significant increase in YFP MFI. A similar effect was observed for the PTM K5.2, targeting intron 5. The addition of the Twister ribozyme to the 5′ end improved *trans*-splicing efficiency, as indicated by an increase in the percentage of YFP^+^ cells and YFP MFI in flow cytometry (Figures 6A and S9B). Additionally, a combination of either 5′ Twister or 5′ HDV PTM K5.2 with asRNAs significantly increased *trans*-splicing efficiency, observed in both YFP^+^ cells and YFP MFI (Figures 6A and S9B). In contrast, expression of K6.5 under the CMV promoter, with ribozyme addition, did not affect *trans*-splicing efficiency (Figures 6A and S9C). Furthermore, results obtained for K2.3 and K5.2 were validated by western blot analysis. Western blot analysis confirmed a significant increase in YFP signal for PTM K2.3 expressed under CMV with attached 5′ HDV. In contrast, PTM K5.2 expressed under CMV showed no significant change in YFP signal when either the 5′ HDV or 5′ Twister ribozyme was attached. Furthermore, we did not detect a significant difference in target intron reporter protein expression (Figures 6B and 6C).

As we observed an improved *trans*-splicing in both cases when K2.3 was expressed under either the U6 or CMV promoter with HDV and Twister ribozymes placed at the 5′, we further compared these two K2.3 variants. Flow cytometry results showed that 5′ HDV K2.3 expressed under the CMV promoter increased *trans*-splicing efficiency, whereas the 5′ Twister K2.3 exhibited similar efficiency under both promoters. Notably, the combination of 5′ Twister K2.3 expressed from the U6 promoter with asRNA BP4 resulted in significantly higher *trans*-splicing efficiency in YFP^+^ cells compared with expression under the CMV promoter (Figures S10A and S10B). Western blot analysis, however, showed no significant difference between the variants (Figures S10C–S10E). Given that K2.3 with 5′ HDV expressed under the CMV promoter demonstrated the best *trans*-splicing efficiency, it was selected for further experiments.

### *Trans*-splicing of endogenous *CTNNB1*

Based on the split YFP reporter screening for the efficient *trans*-splicing targeting CTNNB1 introns 2, 5, and 6, we selected the best-performing PTMs to test their ability to induce endogenous CTNNB1 *trans*-splicing in HEK293T cells. The investigated candidates were CMV:5′HDV:K2.3 and CMV:5′Twister: K5.2, targeting introns 2 and 5, respectively. For intron 6, since adding ribozymes or expressing under the CMV promoter did not improve the efficiency of U6:K6.5, we selected the unmodified version of this PTM for further testing.

**Figure 7:**
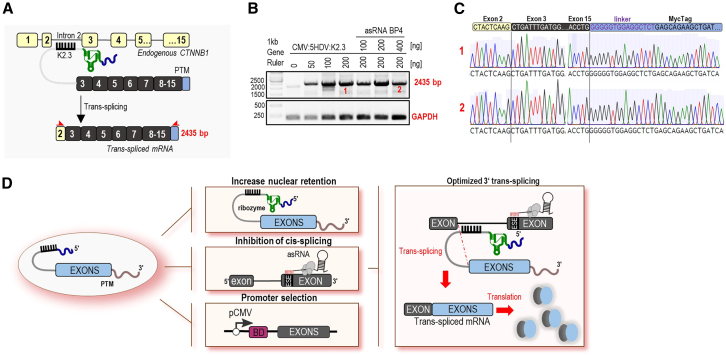
Endogenous detection of *trans*-splicing efficiency in HEK293T cells (A) Schematic example of best-performing PTM K2.3 with coding *CTNNB1* region targeting endogenous *CTNNB1* intron 2. The result of *trans*-splicing is *trans*-spliced *CTNNB1* mRNA that contains the endogenous *CTNNB1* region upstream from the target intron (yellow exons) and the exogenous *CTNNB1* region with myc-tag from the PTM candidate. To detect *trans*-spliced mRNA by using PCR, specific primers (asterisks marked in red) were designed to detect exon 2 and the myc-tag from the PTM K2.3. The size of the band that corresponds to *trans*-spliced mRNA is 2,435 bp. (B) Correctly *trans*-spliced *CTNNB1* mRNA was detected 48 h after transfection by using semi-qPCR. HEK293T cells transfecting with empty vector (pcDNA3) were used as a negative control. (C) Sanger sequencing of the PCR product further confirmed the accuracy of the *trans*-splicing in HEK293T cells. Bands labeled 1 and 2 were excised from the gel and subsequently sent for Sanger sequencing. Data are representative of two independent experiments. (D) Schematic representation of the key optimizations implemented in this study to enhance *trans*-splicing efficiency. The incorporation of 5′ ribozymes into PTMs expressed under the CMV promoter, combined with asRNAs designed to inhibit *cis*-splicing, resulted in an improvement of *trans*-splicing efficiency.

To assess the ability of PTM candidates to induce *trans*-splicing on endogenous *CTNNB1*, the 3′ C-YFP sequence of PTMs was replaced with the corresponding coding sequence of *CTNNB1*, depending on the intron insertion site, with an added sequence for the myc-tag at 3′. We initially transfected HEK293T cells with the most promising optimized PTM candidates for introns 2, 5, and 6, and successful *trans*-splicing resulted in the fusion of endogenous *CTNNB1* pre-mRNA upstream from the target intron with the wild-type coding sequence of *CTNNB1*-myc-tag derived from the PTM (Figures 7A, S11A, and S11B). Forty-eight hours after transfection, total RNA was isolated and reverse transcribed. Semi-qPCR with a specific forward primer for endogenous *CTNNB1* and a specific reverse primer for myc-tag produced *trans*-spliced PCR fragments of the predicted size 2,435 bp for the PTM CMV:5′HDV:K2.3, 1,878 bp for the CMV:5′Twister:K5.2, and 1,575 bp for the PTM U6:K6.5 (Figures 7A, S11A, and S11B). These primers do not generate PCR products from a *cis*-spliced target. The successful specific *trans*-splicing was confirmed for intron 2. Furthermore, *trans*-splicing was detected when asRNAs were added in combination with PTM candidate K2.3 (Figure 7B). The sequence analysis of *trans*-spliced fragments further validated *trans*-splicing (Figure 7C). As a negative control, the BD of PTM CMV:5′HDV:K2.3 was replaced with a random sequence, which resulted in no detectable *trans*-splicing of the endogenous *CTNNB1* gene (Figure S11C). A *trans*-spliced mRNA product of the correct size was initially identified for the CMV:5′Twister:K5.2. However, Sanger sequencing of these bands did not confirm the expected splice junction between exons 5 and 6 (data not shown). Furthermore, specific on-target *trans*-splicing for the U6:K6.5 at the RNA level was not detected (Figure S11B). Instead, a shorter PCR product of approximately 500 bp was observed, suggesting the presence of an unspecific *trans*-splicing event.

## Discussion

Gene therapy strategies like gene replacement therapy, regulation of gene expression with siRNA and ASOs, and CRISPR-Cas genome editing techniques are increasingly entering clinics. There is a pressing need to expand the repertoire of advanced therapies with strategies that can provide safety based on endogenous transcriptional regulation. The mRNA transcript-targeting technique of SMaRT holds the potential to circumvent many of the challenges of other methods, particularly where excessive expression could be toxic. While the SMaRT-based therapy for ABCA4-related retinopathy is currently being evaluated in a clinical trial,^41^ the implementation of this strategy for other targets and improvement of the efficiency of SMaRT are very much needed. Here, we explored the potential of the SMaRT strategy with novel improvements aiming for a therapeutic strategy for the CTNNB1 syndrome. Upregulation of the endogenous *CTNNB1* gene expression up to 2-fold from the remaining functional gene copy should be sufficient to rescue its function, while the gene replacement therapy typically yields much higher upregulation. In some genes, including the neurodevelopmental important *CTNNB1*, *FOXG1*, and *EHMT1*, this is problematic due to their sensitivity to both under- as well as overexpression, which requires their gene expression to be strictly maintained within a narrow window. As the SMaRT strategy does not interfere with the gene regulation but rather corrects the effects of the mutation on a transcriptional level, dosage-sensitive genes such as *CTNNB1* are ideal candidates for its implementation.

The selection of suitable target introns within the *CTNNB1* transcript was guided by 3′ SS strength predictions, which indicated that introns 2, 3, 5, and 6 had lower 3′ SS strength than the PTM, making them ideal candidates for *trans*-splicing. We employed a tiling approach to design PTM with varying BDs across the target introns, which allowed us to assess which BDs provided the highest *trans*-splicing efficiency. This approach revealed the three most promising PTM candidates for introns 2, 5, and 6. It has been reported that the position of PTM binding in the target intron correlates with the *trans*-splicing efficiency. In accordance with other 3′ *trans*-splicing adopting studies,^42^^,^^43^ BDs that target the 5′ part of the target intron showed better *trans*-splicing efficiency, as also exhibited by our best PTM candidates, K2.3 and K6.5. The proximity of the PTM candidate to the 5′ SS likely allows the spliceosome machinery to recognize the 3′ SS of the PTM candidate before the 3′ SS of the target intron, which consequently increases the likelihood of inducing *trans*-splicing. However, this pattern was not observed with PTMs targeting intron 5. In this case, the most effective PTM, K5.2, targets the 3′ region of the intron, covering the branchpoint and 3′ SS. This observation agrees with previous studies,^44^^,^^45^ suggesting that binding closer to the 3′ SS can enhance *trans*-splicing efficiency, particularly in shorter introns like intron 5 (89 nt), where space constraints may limit effective spliceosome assembly when PTMs bind near the 5′ end. In contrast, PTMs K3.1 and K3.2 targeting intron 3 did not exhibit detectable *trans*-splicing activity in the split YFP reporter system. This lack of activity may be due to the lower GC content across the entire BD sequences of K3.1 and K3.2, calculated by the GC Content Calculator.^46^ Both BDs contain a high proportion of adenine and uracil, which likely results in a more flexible RNA structure and weaker base pairing. This could reduce the binding affinity between the PTM and the target pre-mRNA, leading to an unstable RNA-RNA interaction and, ultimately, inefficient *trans*-splicing.^47^ Together, these findings suggest that the optimal binding position for PTMs may vary depending on the structural and sequence features of each target intron. The addition of short asRNAs designed to target 3′SS and ESEs significantly boosted *trans*-splicing efficiency for all tested introns. This enhancement was most notable for intron 5, where the asRNA SC35 increased YFP^+^ cells from 16% to 55%. These findings are in line with previous studies that utilized short synthetic ASOs to inhibit competing *cis*-splicing, thereby promoting the desired *trans*-splicing event.^20^ However, combining multiple asRNAs did not result in further improvement, indicating that a single, well-designed asRNA is sufficient to enhance PTM-mediated *trans*-splicing. The lack of activity observed with multiple asRNAs could be attributed to the formation of unintended RNA secondary structures when co-expressed as a longer RNA from a single vector. The stable secondary structure may interfere with proper folding and reduce the accessibility of the asRNA sequences, thereby preventing efficient binding to their target sites on the pre-mRNA.

The choice of promoter also played an important role in determining the *trans*-splicing efficiency. Expressing PTM K2.3 under the CMV promoter significantly enhanced *trans*-splicing compared with the initial choice of U6 promoter, while K5.2 and K6.5 showed no significant difference between promoters. CMV promoter is utilized by Pol II and results in a capped RNA. We further explored the use of self-cleaving ribozymes at the 5′ and 3′ ends of the PTM molecules. Placement of an active self-cleaving ribozyme at the 5′ end of the PTM K2.3 and PTM 5.2 significantly enhanced *trans*-splicing efficiency at both RNA and protein levels and significantly reduced the leakage of PTM 2.3 into translation, likely due to the removal of the 5′ cap. Ribozymes have thus far been utilized to cleave the polyadenylation tail off the 3′ *trans*-splicing PTMs to facilitate their retention inside the nucleus.^28^ Our findings are the first to suggest that 3′ *trans*-splicing can be analogously enhanced by cleaving the redundant 5′ cap of PTM. Transcripts from both polymerase II and III driving transcription from CMV and U6 promoters, respectively, were reported to possess a 5′ cap (7-methyl guanine^38^ and gamma monomethyl^48^). Accordingly, in our experiments cleaving off the 5′ cap from the PTMs expressed under both promoters significantly enhanced 3′ *trans*-splicing.

Interestingly, the selection of promoter and the addition of ribozymes did not significantly improve the *trans*-splicing efficiency of the unoptimized PTM K6.5, suggesting that the system may have already reached its maximal efficiency during the initial screening.

In addition to flow cytometry, we validated our screening results using western blot analysis. We initially expected that increased PTM-mediated *trans*-splicing efficiency would reduce the HA-tagged *cis*-spliced product from the target intron reporter. However, western blot analysis showed significant increase in the HA-tagged *cis*-spliced product in certain experiments (Figures 5B, 5D, S7B, and S7C). This effect was not consistent across all PTMs. For instance, PTM K2.3 exhibited variable results in independent experiments (Figures 3A, 5B, and S7A) where it was tested alongside other PTMs. We hypothesize that this increase could be due to stabilization of the target intron reporter upon PTM binding, but it is more likely related to overexpression of the reporter itself. Despite this background effect, we consistently detected efficient *trans*-splicing, as indicated by the presence of the myc-tagged YFP, which is produced only through successful *trans*-splicing. These findings suggest that the PTM constructs effectively mediate *trans*-splicing even in the presence of variable background *cis*-splicing.

Finally, combining multiple optimization strategies we successfully demonstrated that PTM targeting intron 2 induces robust on-target *trans*-splicing of the endogenous *CTNNB1* transcripts in HEK293T cells. While the best results were obtained from a combination of PTM with ribozyme and asRNA in separate constructs, future efforts may be able to identify a single construct strategy that could be more robust for therapeutic applications. Moreover, effective *in vivo* delivery will be essential for therapeutic applications, with AAV vectors representing a promising platform due to their efficient, tissue-specific, and sustained expression of *trans*-splicing components.

The experiments in this report focused on *CTNNB1*, although the same design rules are likely to be applicable for other therapeutic targets, where the endogenous physiological regulation needs to be maintained. Currently, the efforts on CTNNB1 therapy are focused on gene replacement therapy, where the tools to prevent excessive expression in the liver have been used and demonstrated safe and effective results in animals.^49^

In conclusion, this study presents an exploration of strategies to optimize PTM-mediated *trans*-splicing for the *CTNNB1* gene. Our findings underscore the importance of combining the selection of the BD, the addition of asRNA to mask *cis*-splicing motifs, the use of CMV promoter, and cleavage of 5′ of the 3′ *trans*-splicing PTM in achieving high efficiency of *trans*-splicing (Figure 7D). These results provide a solid foundation for future applications of *trans*-splicing for the CTNNB1 syndrome, including gene editing and the treatment of genetic disorders.

## Materials and methods

### Plasmid construction

Split YFP target minigenes were cloned into the pcDNA3.1D vector backbone. PTMs were cloned into the pgRNA (Addgene plasmid #44248) or pcDNA3.1D vector backbone. The plasmid encoded with *CTNNB1* (Cat. no. HG11279-CY) was provided by Sino Biological (Beijing, China). Target *CTNNB1* intronic sequences were ordered as gblocks (IDT, Inc., Coralville, IA, USA). PCR amplification was performed using RepliQa HiFi ToughMix (Quantabio, Beverly, MA, USA). All plasmids were constructed using the Gibson assembly method and are provided in Table S2.

### Construction of an asRNA library

ESEfinder and SVM-BPfinder prediction tools were used to predict ESEs sequences and BPs. Sequence for the cassette with U1 promoter and modified U7 snRNA was taken from the literature^32^^,^^33^ and ordered as gblock (IDT, Inc., Coralville, IA, USA) and cloned into pgRNA vector (Addgene plasmid #44248) using the Gibson assembly method. Designed asRNA sequences were ordered as primers and added to U1-U7 cassette using Gibson assembly. Detailed sequences of all asRNA constructs are listed in Table S6.

### Cell culture

HEK293T cells were cultured in DMEM (Thermo Fisher Scientific) supplemented with 10% v/v FBS (Thermo Fisher Scientific). The cell line was maintained in a humidified incubator at 37°C with 5% CO_2_. Cell lines were obtained from the ATCC culture collection.

### Transient transfection

HEK293T cells were washed with phosphate-buffered saline (PBS) and detached from the surface using Trypsin-EDTA solution (Sigma-Aldrich, Cat. no. T3924; St. Louis, MO, USA). The cell concentration was measured using Countess Cell Counting Chamber Slides or EVE Cell Counting Slides kits with Trypan blue as an indicator of live cells and measured on the Countess 3 Automated Cell Counter (Invitrogen, Thermo Fisher Scientific). For RNA extraction, immunoblotting, and flow cytometry, 1 × 10^5^ cells per well were seeded in 24-well TPP plates. At 30%–50% confluence, HEK293T cells were transfected with a mixture of DNA and PEI (6 μL per 500 ng of DNA, stock concentration 0.324 mg/mL, pH 7.5). The PEI stock concentration was diluted in 150 mM NaCl and mixed at a 1:1 ratio with the appropriate DNA, also diluted in 150 mM NaCl. This was incubated at room temperature for 15 min and added to the cell media in plates. The amounts of transfected plasmids are listed in Table S7. For cytometry experiments, plasmid expressing iRFP was added to transfection mixes as a transfection control.

### Flow cytometry

The transfected HEK293T cells were maintained at 37°C in a 5% CO_2_ environment. At 48 h post-transfection, the cells were prepared in PBS for analysis on the Cytek Aurora Flow Cytometry System (Cytek Biosciences, Fremont, CA, USA) with SpectroFlo software. For flow cytometry compensation and gating controls, we used non-transfected HEK293T cells as negative controls and constitutive expression vectors encoding YFP, tagBFP, and iRFP fluorescent proteins as single stain controls. Cells were first gated for singlets, then the expression of iRFP as a transfection control, and then for the expression of YFP. Additional experiments conducted during the revision phase were performed using the Attune NxT Flow Cytometer (Thermo Fisher Scientific) with Attune NxT Software. The same compensation strategy and gating approach were applied to ensure consistency across instruments. The gating strategy used in all experiments is described in Figure S3.

### RNA isolation and cDNA synthesis

At 48 h, total RNA was extracted from transfected HEK293T using the High Pure RNA Isolation Kit (Roche, Basel, Switzerland) according to the manufacturer’s protocol. Reverse transcription was performed on 1 μg of total RNA using a high-capacity complementary DNA (cDNA) reverse transcription kit (Applied Biosystems, Waltham, MA, USA) with a mixture of random oligonucleotides, according to the manufacturer’s instructions.

### Semi-qPCR

To detect *trans*-splicing efficiency at the RNA level, semi-qPCR amplification was performed on the cDNA using RepliQa HiFi ToughMix (Quantabio). PCR bands were analyzed via gel electrophoresis on a 1% agarose gel (Zellbio, Lonsee, Baden-Württemberg, Germany), extracted and purified by peqGOLD gel extraction kit (VWR Peqlab, Darmstadt, Germany), and analyzed by Sanger sequencing. Detailed sequences of primers used for semi-qPCR are listed in Table S8**.**

### Western blot analysis

The cells were washed with PBS and lysed in 100 μL of 1× Passive lysis buffer (Promega, Madison, WI, USA). Total protein concentration in the supernatant was measured by the bicinchoninic acid (BCA) method. Samples were denatured by 5-min incubation at 95°C in SDS with a reducing agent. We then loaded 30–50 μg of total protein per sample onto the SDS-PAGE gel (Bio-Rad, Hercules, CA, USA) with PageRuler Plus Prestained Protein Ladder (Thermo Fisher Scientific) as size standard. SDS-PAGE was run under denaturing conditions at 200 V for 60 min. Proteins were transferred to a Hybond ECL nitrocellulose membrane (GE Healthcare, Chicago, IL, USA). After protein transfer, membranes were blocked in 0.2% (w/v) iBlock (Thermo Fisher Scientific) for 1 h at room temperature. Following blocking, membranes were incubated overnight with primary antibodies diluted in 0.2% (w/v) iBlock. The next day, membranes were washed three times with PBS-T and then incubated with secondary antibodies diluted in 0.2% (w/v) iBlock for 1 h at room temperature. Membranes were washed three times with PBS-T before protein detection. Myc-tagged *trans*-spliced YFP was specifically detected with primary antibodies mouse anti-myc (Cell Signaling Technology, Cat. no. 2276S) at 1:1,000 and secondary antibodies Goat anti-mouse-HRP (Jackson ImmunoResearch, Cat. no. 115-035-003) at 1:2,000 ratios. HA-tagged target intron reporter was specifically detected with primary antibodies Rabbit anti-HA-tag (Sigma-Aldrich, Cat. no. H6908) at 1:1,000 and secondary antibodies Goat anti-rabbit-HRP (Thermo Fisher Scientific, Cat. no. 65–6120) at 1:2,000 ratios. For loading control, we detected β-actin using antibodies mouse anti-β-actin (Cell Signaling Technology, Cat. no. 3700) at 1:1,000 and secondary antibodies Goat anti-mouse-HRP at 1:3,000 ratios or α/β tubulin protein using primary antibodies Rabbit Alpha/Beta Tubulin (Cell Signaling Technology, Cat. no. 2148) at 1:1,000 ratios and secondary antibodies Goat anti-rabbit-HRP at 1:2,000 ratios. Detection of HRP was achieved by incubation of the membrane with SuperSignal West Pico or Femto Maximum Sensitivity Substrate (Thermo Fisher Scientific). The immunoblots were visualized on a G-box membrane that was imaged with the G:Box Chemi XT 4 Chemiluminescence and Fluorescence Imaging System (Syngene, Bangalore, Karnataka, India).

Uncropped images of blots are available in Figures S12–S17. Protein levels were quantified using ImageJ. Band intensities were first normalized to the loading control, followed by normalization to the reference sample, as specified in the figure legends.

### Confocal microscopy

Co-transfection of PTM and asRNA was performed in eight-well IBIDI plates for 48 h. Microscopic images of cells were taken by a Leica TCS SP5 inverted laser-scanning microscope on a Leica DMI 6000 CS module equipped with an HCX Plan-Apochromat lambda blue 63× objective, numerical aperture 1.4 (Leica Microsystems, Wetzlar, Germany). A 488-nm laser line of a 100-mW argon laser with 10% laser power was used for the detection of YFP, where emitted light was detected between 500 and 600 nm. For acquisition and image processing we used Leica LAS AF program (Leica Microsystem).

### qPCR

Co-transfection of PTM and asRNA into HEK293T was performed in 24-well format for 48 h. On RNA isolation and reverse transcription to cDNA, we determined the efficiency of *trans*- and *cis*-splicing using qPCR. To detect *trans*-splicing efficiency, primers were designed to amplify the 265-bp-long segment between N-YFP and myc-tag that is assembled only in the *trans*-spliced PTM. To detect *cis*-splicing, primers were designed to amplify the segment between N-YFP and the exon of the coding region that follows each target intron (Figure 3B). A white 96-well LightCycler 480 Multiwell Plate was used for qPCR reaction with each reaction containing 1X SYBR Green I Master Mix (Roche), 20 ng of cDNA, and 0.5 μM of forward and reverse primer and was run in LightCycler 480 instrument (Roche). As a control of the endogenous gene expression we amplified a short segment of GAPDH. All measurements were performed in duplicate or triplicate. Relative change in RNA expression was calculated using the 2-ΔΔCq method. Detailed sequences of primers used for qPCR are listed in Table S8.

### TopFlash luciferase reporter assay

TopFlash luciferase reporter assay utilizes a TopFlash plasmid (Addgene; #12456) containing Firefly luciferase, which is regulated by a promoter that binds TCF/LEF transcription factors, key components of the Wnt signaling pathway. A plasmid FopFlash (Addgene #124578) containing mutations within the TCF/LEF binding sites was used as a control. HEK293T cells were co-transfected with PTM candidates, either in the presence or absence of the target reporter system, along with the either TopFlash or FOPFlash plasmid. To control for transfection efficiency, cells were also co-transfected with the phRL-TK plasmid (Promega), which constitutively expresses Renilla luciferase. Both Firefly luciferase and Renilla luciferase activities were measured using the dual luciferase assay (Promega) on a Centro LB 963 microplate reader (Berthold Technologies, Bad Wildbad, Germany) with Simplicity 4.2 software. Relative luciferase units (RLUs) were calculated by normalizing the Firefly luciferase activity to the Renilla luciferase activity in the same sample. Amounts of plasmid used in this assay are provided in Table S7.

### Statistics

Results were analyzed in Excel and presented in GraphPad Prism (GraphPad, Boston, MA, USA). Statistical significance was determined by the two-way or ordinary one-way ANOVA with Dunnett’s multiple comparisons test, and Student’s t test. Results with *p* value <0.05 were deemed statistically significant.

## Data availability

All data supporting the findings of this study are available within the article or supplemental information. Additional data are available from the corresponding author upon reasonable request.

## Acknowledgments

This work was funded by the 10.13039/501100004329Slovenian Research Agency (grants Z1-3193 (to P.S.-L.), J7-4537, P4-0176, ARIS-GRAVITACIJA-STRATESKI-2024/12), and the CTNNB1 Foundation. The authors thank Dr. Damjan Osredkar for valuable comments and Tina Hertiš for technical assistance.

## Author contributions

M. Maruna conceptualized the study, designed and performed the experiments, analyzed and visualized the data, and wrote the manuscript. P.S.-L. conceptualized the study, designed and performed the experiments, analyzed the data, wrote the manuscript, and obtained funding. M. Meško performed the experiments. Š.M. reviewed and revised the manuscript before resubmission. R.J. conceptualized and supervised the study, edited the manuscript, and obtained funding.

## Declaration of interests

The authors declare no competing interests.

## Supplemental information

Document S1. Figures S1–S17 and Tables S1–S8

Document S2. Article plus supplemental information
