# Supplementary material for: RNA trans-splicing to rescue β-catenin: A novel approach for treating CTNNB1-Haploinsufficiency disorder
Source: Mol Ther Nucleic Acids. 2025 Aug 12;36(3):102680. doi: 10.1016/j.omtn.2025.102680 (PMC12398835; doi:10.1016/j.omtn.2025.102680)
Supplement: Document S1. Figures S1–S17 and Tables S1–S8 [file mmc1.pdf]

OMTN, Volume *36*

Supplemental information

RNA *trans*-splicing to rescue β-catenin: A novel approach for treating CTNNB1-Haploinsufficiency disorder

Matea Maruna, Petra Sušjan-Leite, Maja Meško, Špela Mirošević, and Roman Jerala

A

gtttgtgtcattaaatctttagttactgaattggggctctgcttcgttgccattaagccagtctggctgagatccccctgctttcctctctccct

gcttacttgtcaggctaccttttgctccattttctgctcactcctcctaatggcttggtgaaatagcaaacaagccaccagcaggaatctag

tctggatgactgcttctggagcctggatgcagtaccattcttccactgattcagtgagtaactgttaggtggttccctaagggattaggtatt

tcatcactgagctaaccctggctatcattctgcttttcttggctgtctttcagatttgactttatttctaaaaatatttcaatgggtcatatcac

agattcttttttttaaattaaagtaacatttccaatctactaatgctaatactgtttcgtatttatagCTGATTTGATGGAGTTGGAC

ATGGCCATGGAACCAGACAGAAAAGCGGCTGTTAGTCACTGGCAGCAACAGTCTTACCTGGACTCTGGA

ATCCATTCTGGTGCCACTACCACAGCTCCTTCTCTGAGTGGTAAAGGCAATCCTGAGGAAGAGGATGTGG

ATACCTCCCAAGTCCTGTATGAGTGGGAACAGGGATTTTCTCAGTCCTTCACTCAAGAACAAGTAGCTG

K2.1 **K2.2** **K2.3** **K2.4** **K2.5** **K2.6** **K2.7** **K.I2(rv)**

B

Gtaagagtattatttttcattgccttactgaaagtcagaatgcagttttgagaactaaaaagttagtgtataatagtttaaataaaatgttg

tggtgaagaaaagagagtaatagcaatgtcacttttaccatttaggatagcaaatacttaggtaaatgctgaactgtggatagtgagtgt

tgaattaaccttttccagATATTGATGGACAGTATGCAATGACTCGAGCTCAGAGGGTACGAGCTGCTATGTTCC

CTGAGACATTAGATGAGGGCATGCAGATCCCATCTACACAGTTTGATGCTGCTCATCCCACTAATGTCCAG

CGTTTGGCTGAACCATCACAGATGCTGAAACATGCAGTTGTAAACTTGATTAACTATCAAGATGATGCAG

K3.1 **K3.2**

C

GtaagaaaacatgtcagaatgcttgaagctaaaaagtagaagagtatactcacaatatttctgatgaggctttttttcttcttcccagTTC

ACCAGTGGATTCTGTGTTGTTTTATGCCATTACAACTCTCCACAACCTTTTATTACATCAAGAAGGAGCTA

AAATGGCAGTGCGTTTAGCTGGTGGGCTGCAGAAAATGGTTGCCTTGCTCAACAAAACAAATGTTAAAT

TCTTGGCTATTACGACAGACTGCCTTCAAATTTTAGCTTATGGCAACCAAGAAAGCAAG

K5.1 **K5.2**

Figure S1: **Position of PTM binding domains targeting CTNNB1 introns 2, 3, and 5.** (A-D) Uppercase letters represent human CTNNB1 exon 3 (A), 4 (B), and 5 (C), lowercase letters represent the CTNNB1 target introns 2 (A), 3 (B), and 5 (C). The region of intron that is target for particular PTM candidate is underlined and color-coded.

A

Ataagagaattattctttatgtggttttcatggagcattggacacctccagtgtcatgtcattccatgcagtgttcctaacctttttggcaccagg

gaccagtttcgtggaaaacagtttttccatgaatgggttgtgggaatggtttctggatgacaccattccacctcagataatcaggcattagatt

ctcatagggagcgtgcagcctagatccctcgcatgtgcagtccacactagggtttctactcctatgagactctcatggtgcagttgatctgaca

ggaggtagagctcaagccaggtaatgctcgctcacctgccacttacctcctgctgtgcagcccagttcatttctgttcttttaaatttttgagttt

ccatatgtaaagcactatgcgaagtagtagggatatggtaggcaagcttctcttcacacttttgttcttaggtgggatgtagatgttgggaata

ataacctaatatttaatttgtgtagtgggaagaagtggggctatgagggcacataacacaagttgaaactgactctttttgagggttcaagga

gacctcttggaggaagtgatagttgagttcagtgttcaaggatgagaagggattcactaggtgaaggttaggtgagaaaacaacatctttga

aacgaaggaaggagatggaaagttttgggaatttaagaaatactaatagtaaggaggaagaaaggtttgaggtgaggctattgagataga

cttagcagatctcatagggctttgtagagcatgtttaaaagcacaatgggaaatttcagcagaagcctgaaatgatgaaatttgtttttagaa

aattggggcagtgttgaaagggaagatatacagggaatgaaaggacaagcatgaatgatcattttatggtatctgtttttaaggtggatata

attaggaaaattaaagggccaaatgatgaggagttaagtgccagttctggttcaaattttcagtgaatcagttttgatataactttcatcttag

ggcattactcttgcctaccaacatagtttctaaattttttcttttggtgtgatcactgtgggaagaaggaaattgggcccaaactgatacattg

tttggaggactgggatgtctgaatttgagtggaatgctttaaaggacaagttggatagggccccagtatgggggtctgagtgatggggtcc

aggaatacatttaggtccaatggcaagctggctgaaattcttgtataataaaataggttggtaatatggctcttctcagacatgtgatcaaga

ttccttgactaacaagatatatatatatatctttctagCTCATCATACTGGCTAGTGGTGGACCCCAAGCTTTAGTAAATATA

ATGAGGACCTATACTTACGAAAAACTACTGTGGACCACAAGCAGAGTGCTGAAGGTGCTATCTGTCTGCTCT

AGTAATAAGCCGGCTATTGTAGAAGCTG

K6.1 K6.2 K6.3 K6.4 K6.5 K6.6 K6.7 K6.8 K6.9 K6.10 K6.11
K6.12 K6.13

Figure S2: **Position of PTM binding domains targeting CTNNB1 intron 6.** (A) Uppercase letters represent human CTNNB1 exon 6, lowercase letters represent the CTNNB1 target intron 6. The region of intron that is target for particular PTM candidate is underlined and color-coded.

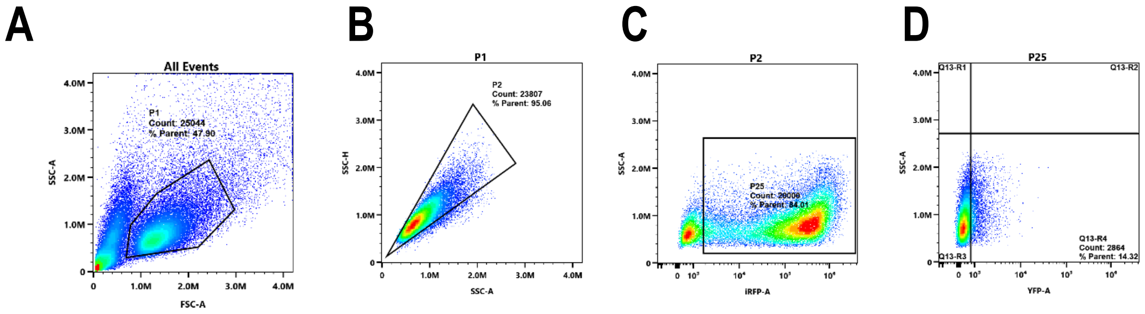

Figure S3: **Flow cytometry gating strategy to detect YFP expression.** (A) Exclusion of debris and dead cells from analysis. (B) Doublets were excluded from the analysis by plotting FSC height vs. FSC area. (C) Cells were then gated for the 20 000 iRFP^{+} cells that presents control of transfection. (D) 20000 of iRFP^{+} cells were further analyzed for the YFP expression that presents successful trans-splicing.

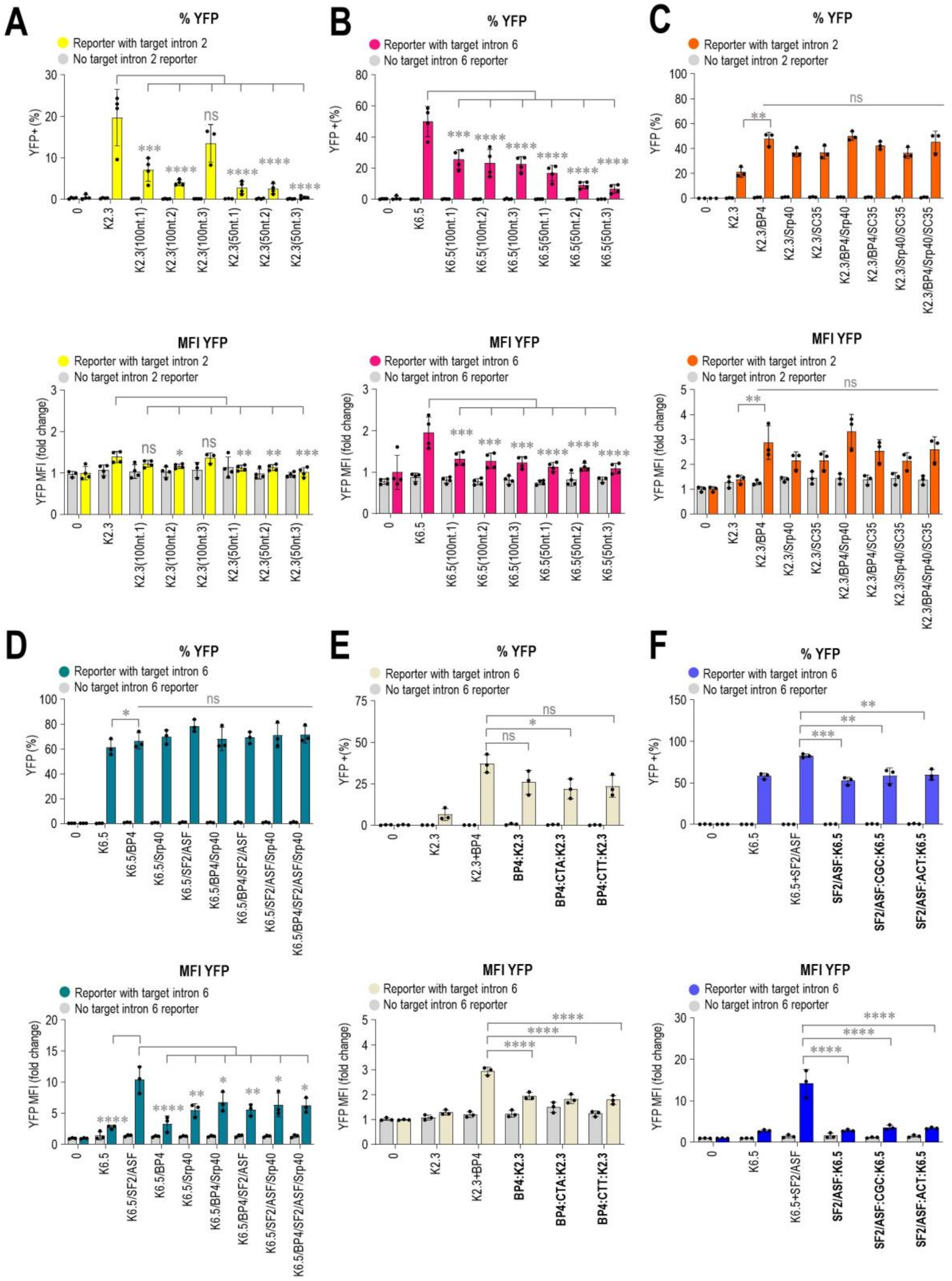

A
% YFP
Reporter with target intron 2
No target intron 2 reporter
MFI YFP
B
Reporter with target intron 6
No target intron 6 reporter
C
D
E
F

Figure S4: **Optimization of best PTM candidates.** (A, B) Effect of shortened binding domain of best preforming PTM K2.3 (A) and K6.5 (B) on trans-splicing measured by flow cytometry. Results are presented as percentage of YFP^+ cells and YFP MFI normalized to negative control – target intron reporter transfected alone. (C,D) Cytometrical analysis of trans-splicing efficiency when two or three best-preforming asRNAs expressed from single vector were combined with best PTM K2.3 (C) for target intron 2 and PTM K6.5 (D) for target intron 6. Results are presented as percentage of YFP^+ cells and YFP MFI normalized to negative control – target intron reporter transfected alone (0). (E,F) Testing of combined PTM K2.3 (E) and 6.5 (F) for the trans-splicing efficiency on flow cytometry. Bars with bold text present PTM K2.3 combined with asRNAs BP4 and K6.5 combined with asRNA SF2/ASF. Results are shown as percentage of YFP^+ cells and YFP MFI normalized to the negative control – target intron reporter transfected alone (0). (A-F) Data are presented as the mean value ± SD from at least three independent experiments. Comparison between tested groups was analyzed using one-way ANOVA with Dunnett's multiple comparison test. ****$p < 0.0001$; ***$p < 0.001$; **$p < 0.01$; *$p < 0.05$; non-significant (ns).

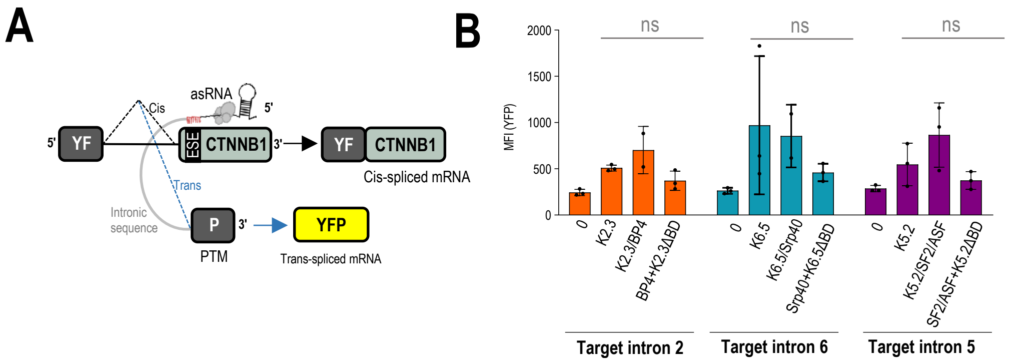

Figure S5: **Optimization of best PTM candidates K2.3, K5.2, and K6.5.** (A) Schematic representation of single-vector trans-splicing RNA constructs in which asRNA sequences (BP4, SRP40, and SF2/ASF) were fused to their respective coding exons in the absence of a binding domain (BD). (B) Flow cytometric analysis of trans-splicing efficiency following transfection with single-vector trans-splicing RNA constructs and corresponding target intron reporter. Data are shown as YFP MFI. Data are presented as the mean value ± SD from at least three independent experiments. Comparison to PTM transfected with target intron reporter was analyzed using one-way ANOVA with Dunnett's multiple comparison test. Non-significant (ns).

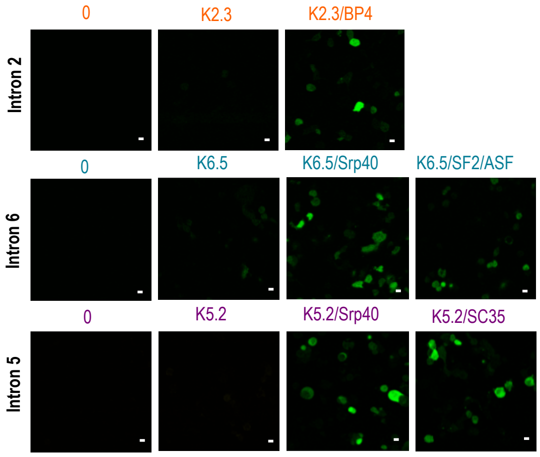

Figure S6: **Detection of efficient trans-splicing using confocal microscopy.** YFP fluorescence upon co-transfection of the best PMT candidates, corresponding asRNA and target intron reporter captured by the confocal microscopy. Images are representative of two independent experiments. Bar represents 10 µM.

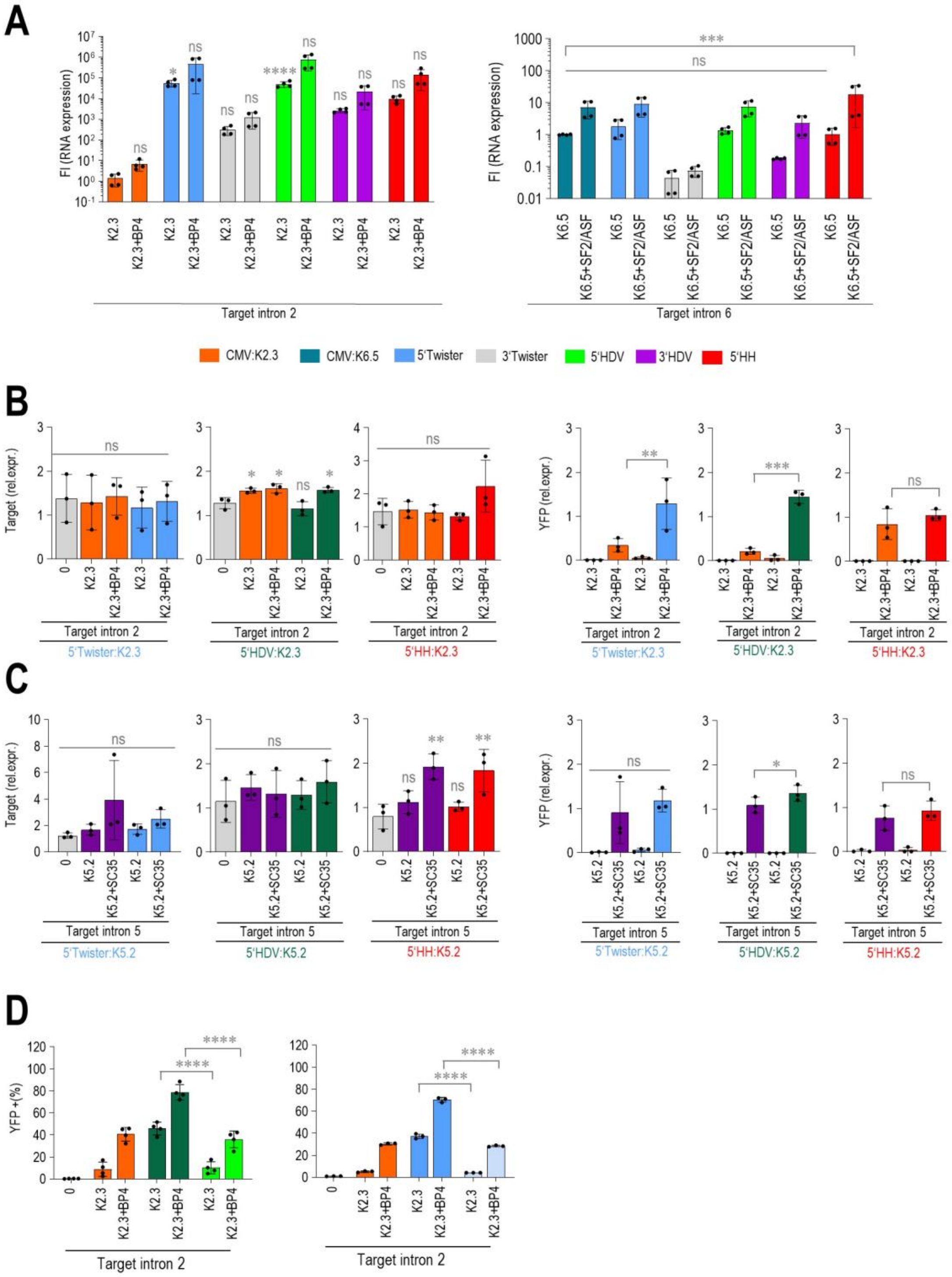
A
FI (RNA expression)
Target intron 2
Target intron 6
CMV:K2.3
CMV:K6.5
5'Twister
3'Twister
5'HDV
3'HDV
5'HH
B
Target intron 2
5'Twister:K2.3
5'HDV:K2.3
5'HH:K2.3
C
Target intron 5
5'Twister:K5.2
5'HDV:K5.2
5'HH:K5.2
D
YFP +(%)
Target intron 2

Figure S7: **Addition of ribozymes HDV, HH and Twister at 5' and 3' end of PTM candidates.** (A) Detection of trans-splicing efficiency when HDV, HH and Twister ribozymes were added at 5' and 3' end of PTM K2.3 and K6.5 by using qPCR. Data are presented as the mean value ± SD of three independent experiments. (B, C) Quantitation of Western blot analysis for the PTM K2.3 (A) and PTM K5.2 (B) was conducted by normalizing myc-tag (YFP) levels to β-actin and subsequently to HA-tag (target). HA-tag levels were also normalized to β-actin. Data from three biological replicates are presented as mean values ± SD. Samples with no detectable band signal were quantified as zero, indicating the absence of detectable protein expression. All uncropped blots used for analysis are available in Figure S13-14. (D) Testing of mutated HDV ant Twister ribozyme added at 5' end of PTM K2.3 by using flow cytometry. Negative control included target intron reporter transfected alone (0). Data are shown as percentage of YFP^+ cells. Data are presented as the mean value ± SD of at three independent experiments. (A-D) Statistical comparisons between tested groups were performed using one-way ANOVA with Dunnett's multiple comparison test. Significance levels are indicated as follows: ****p < 0.0001; ***p < 0.001; **p < 0.01; *p < 0.05; ns, non-significant.

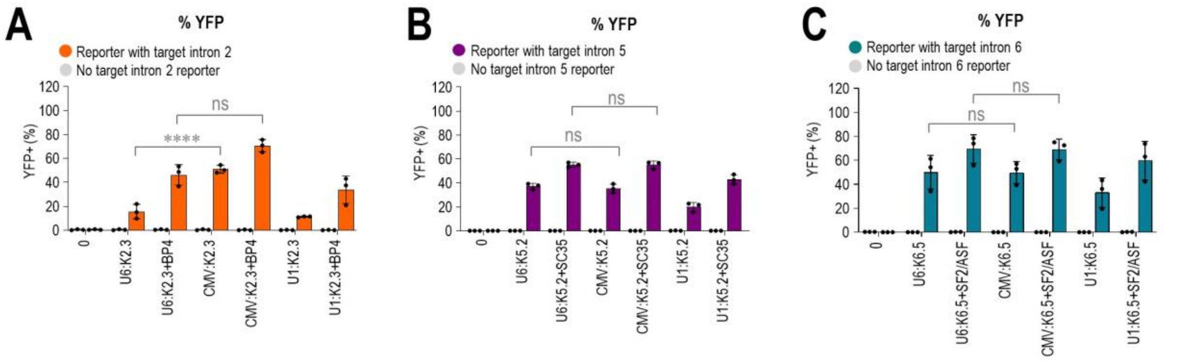

Figure S8: **Testing of different promoters for the efficient trans-splicing.** A) Expression of best candidates K2.3 (A), K5.2 (B) and K6.5 (C) under different promoters. Results are detected by using flow cytometry and shown as percentage of YFP$^+$ cells. Negative controls included target intron reporter transfected alone (0), PTM candidate transfected alone, and PTM with asRNA transfected alone. Data are presented as the mean value ± SD of three independent experiments. Comparison between tested groups was analyzed using one-way ANOVA with Dunnett's multiple comparison test. ****p < 0.0001; non-significant (ns).

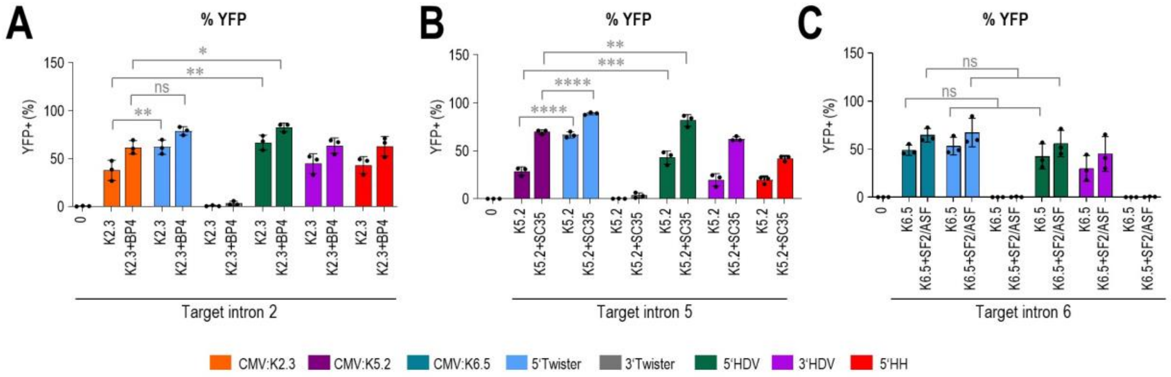

Figure S9: **Addition of ribozymes HDV, HH and Twister at 5' and 3' end of CMV-expressed PTM candidates.** Detection of trans-splicing efficiency when HDV, HH and Twister ribozymes were added at 5' and 3' end of CMV-expressed PTM K2.3 (A), K5.2 (B) and K6.5 (C) using flow cytometry. Trans-splicing efficiency is shown as the percentage of YFP^+ cells. Negative control included target intron reporter transfected alone (0). Data are presented as the mean value ± SD of three independent experiments. Comparison between tested groups was analyzed using one-way ANOVA with Dunnett's multiple comparison test. ****p < 0.0001; ***p < 0.001; **p < 0.01; *p < 0.05; non-significant (ns).

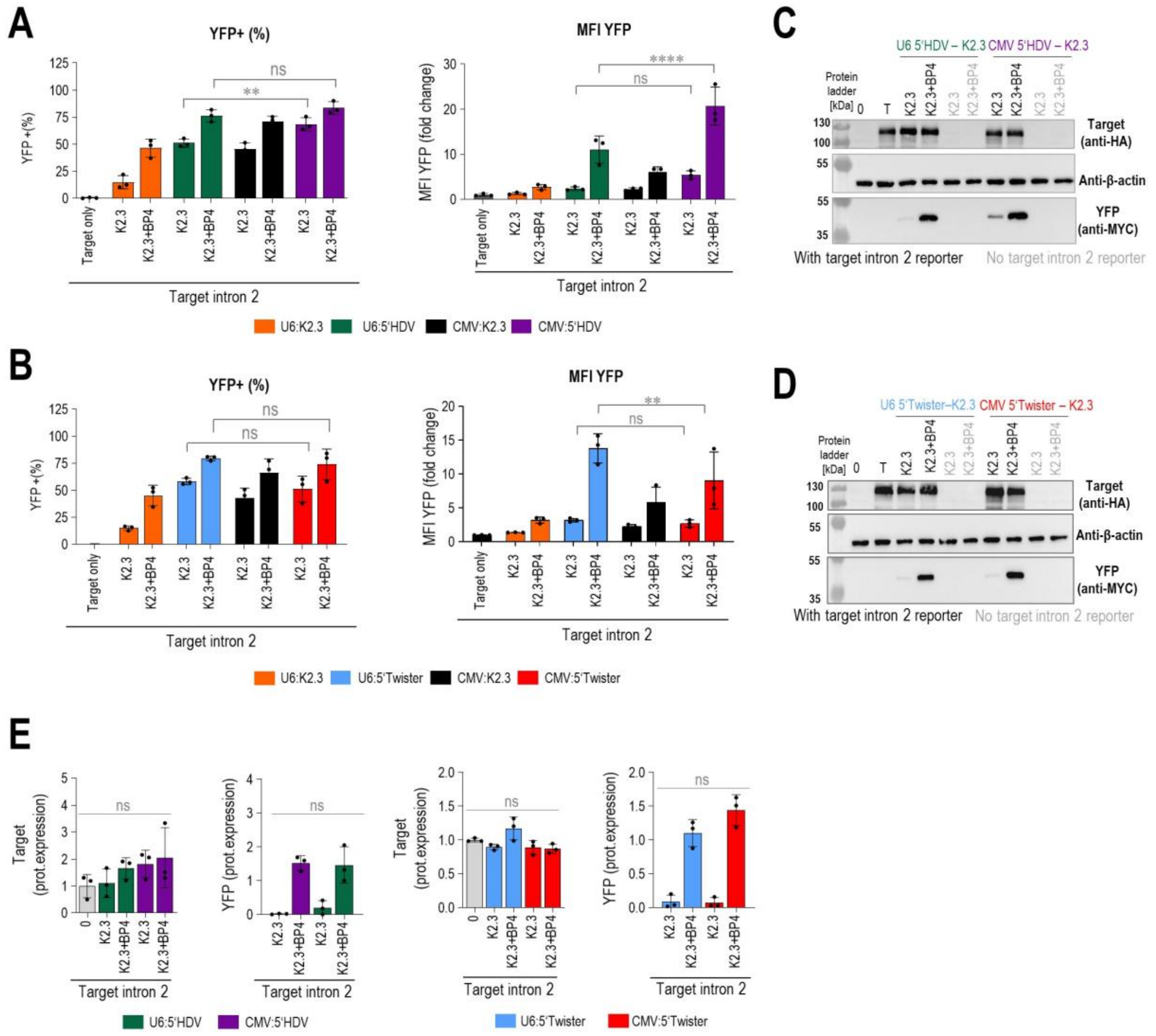

Figure S10: **Comparison of trans-splicing efficiency between PTM K2.3 with 5' HDV and Twister expressed under either U6 or CMV promoter.** (A, B) Comparison of PTM K2.3 expressed under CMV and U6 promoters with HDV (A) or Twister (B) added on 5' end of PTM candidate. Trans-splicing was detected as YFP expression with flow cytometry. Results are shown as percentage of YFP^{+} cells or YFP MFI normalized to target intron reporter transfected alone (0). Data are presented as the mean value ± SD from at least three independent experiments. (C, D, E) Detection of trans-splicing efficiency by western blot analysis using the corresponding anti-myc, anti-HA and β-actin antibodies (C, D), and quantitation of YFP and target intron reporter protein levels (E). (C, D) 0 represents negative control – cells transfected with an empty plasmid vector (pcDNA3); T represents negative control – target intron reporter transfected alone; PTM K2.3 and asRNA BP4 transfected with target intron reporter are marked in black; PTM K2.3 and asRNA BP4 transfected without target intron reporter are marked in grey. Data are representative of three independent experiments. (E) Data from three biological replicates are presented as mean values ± SD. Samples with no detectable band signal were quantified

as zero. Myc-tag (YFP) levels were normalized to β-actin and subsequently to HA-tag (target). HA-tag levels were also normalized to β-actin. All uncropped blots used for analysis can be found in the Figure S17. (A, B, E) Statistical comparisons between tested groups were performed using one-way ANOVA with Dunnett's multiple comparison test. Significance levels are indicated as follows:$^{****}p < 0.0001$; $^{**}p < 0.01$; ns, not-significant.

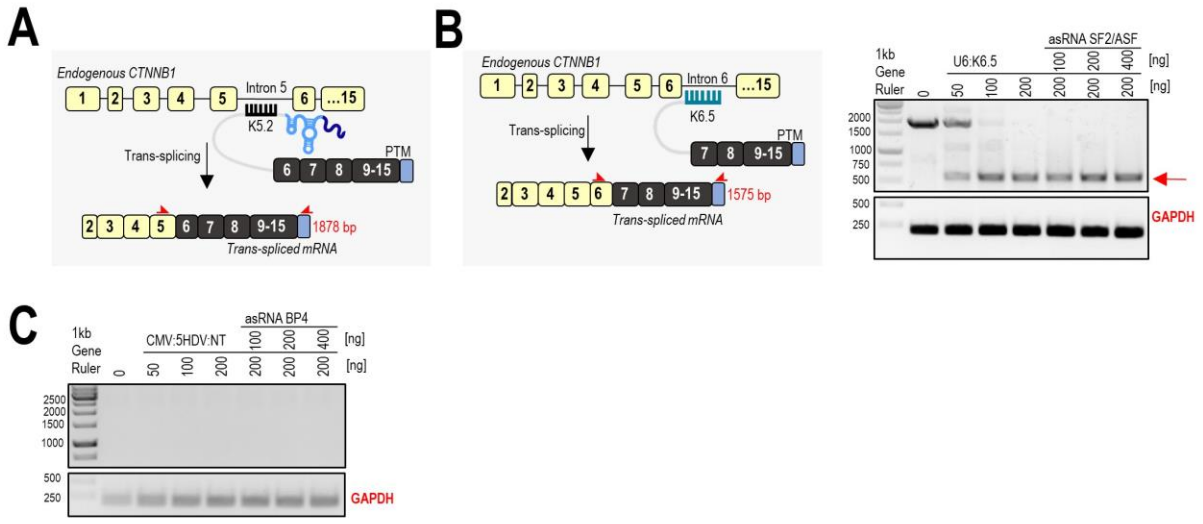

Figure S11: **Detection of trans-splicing in HEK293T cells.** (A, B) Schematic presentation of K5.2 (A) and K6.5 (B) with CTNNB1 coding region targeting endogenous CTNNB1 introns 5 and 6. (B) Testing of K6.5 for the endogenous trans-splicing in HEK293-T cells. RNA was isolated 48h after transfection and performed reverse transcription. cDNA was amplified using semi-qPCR with specific primers targeting endogenous exon 5 and myc-tag from the PTM. PCR product were run on 1% agarose gel. Red arrow presents shorter 500 bp long PCR product. (D) Testing of CMV:5HDV:K2.3 construct with a random (NT) binding domain (CMV5HDV:NT) for the endogenous trans-splicing efficiency.

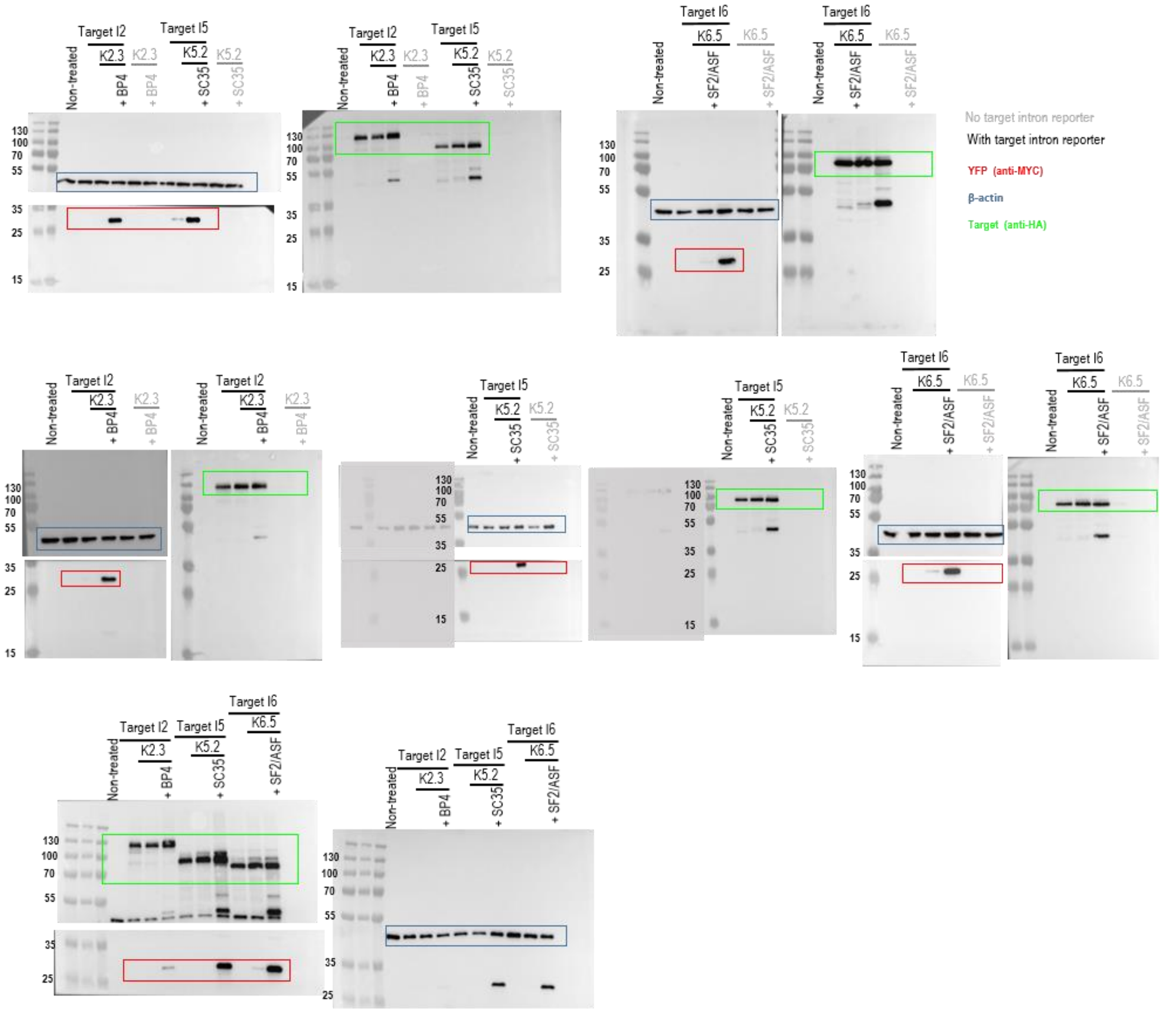

Figure S12: **Uncropped Western blots for generating Figure 3A.** The experiment was performed in three biological replicates. For the first two replicates, samples for the YFP (myc-tag) and β-actin were loaded on the same gel, while target intron reporter (HA-tag) was loaded on different gel. For the third replicate, samples for the YFP (myc-tag), β-actin and target intron reporter (HA tag) were loaded on the same gel. Third replicate was first probed with the YFP (myc-tag) and β-actin, and then target (HA-tag). The parts that are transparently covered are not shown in the present study. Composite images are presented to show the protein marker.

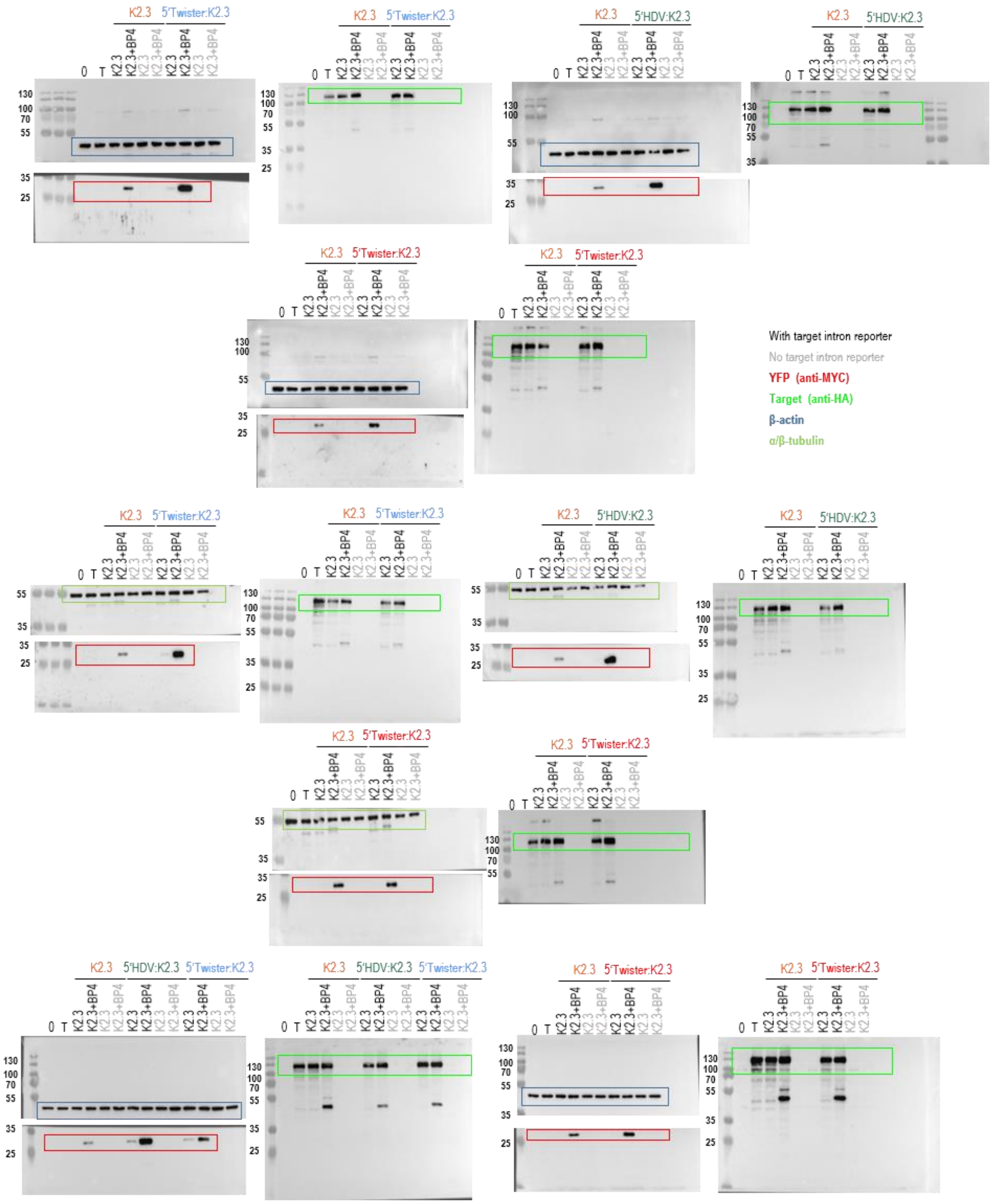

Figure S13: **Uncropped Western blots for generating Figure 4C and S7B**. The experiment was performed in three biological replicates. Cell lysates for the YFP (myc-tag) and β-actin were loaded on the same gel, while target intron reporter (HA-tag) was loaded on different gel. Composite images are presented to show the protein marker.

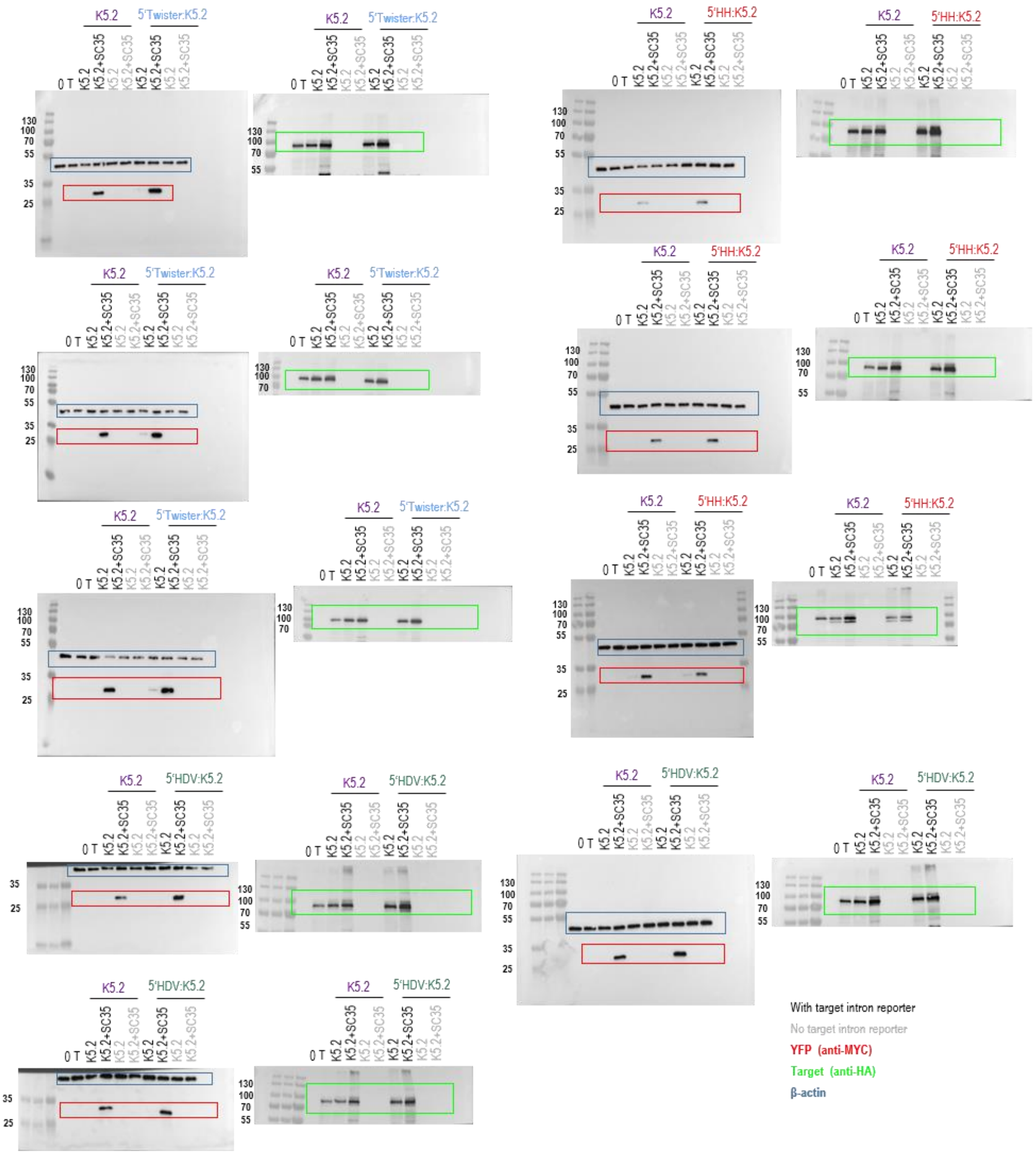

Figure S14: **Uncropped Western blots for generating Figure 4C and S7C**. The experiment was performed in three biological replicates. Cell lysates for the YFP (myc-tag), β-actin and target intron reporter (HA-tag) were loaded on the same gel. Membrane was first probed with the YFP (myc-tag) and β-actin, and then target intron reporter (HA-tag). Composite images are presented to show the protein marker.

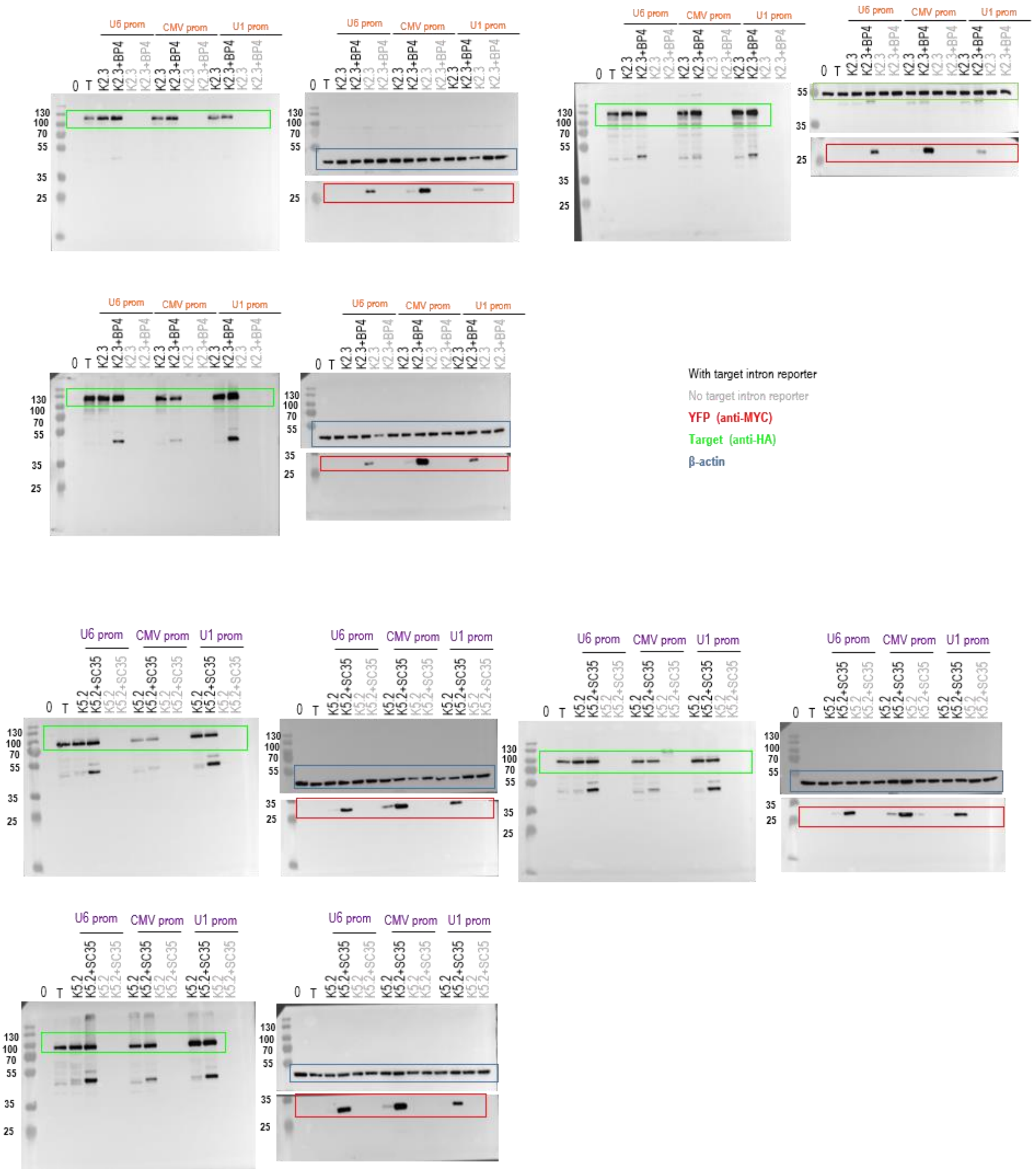

Figure S15: **Uncropped Western blots for generating Figure 5B and 5D**. The experiment was performed in three biological replicates. Cell lysates for YFP (myc-tag) and β-actin were run on the same gel, whereas the target intron reporter (HA-tag) was run on a separate gel. Composite images are presented to show the protein marker.

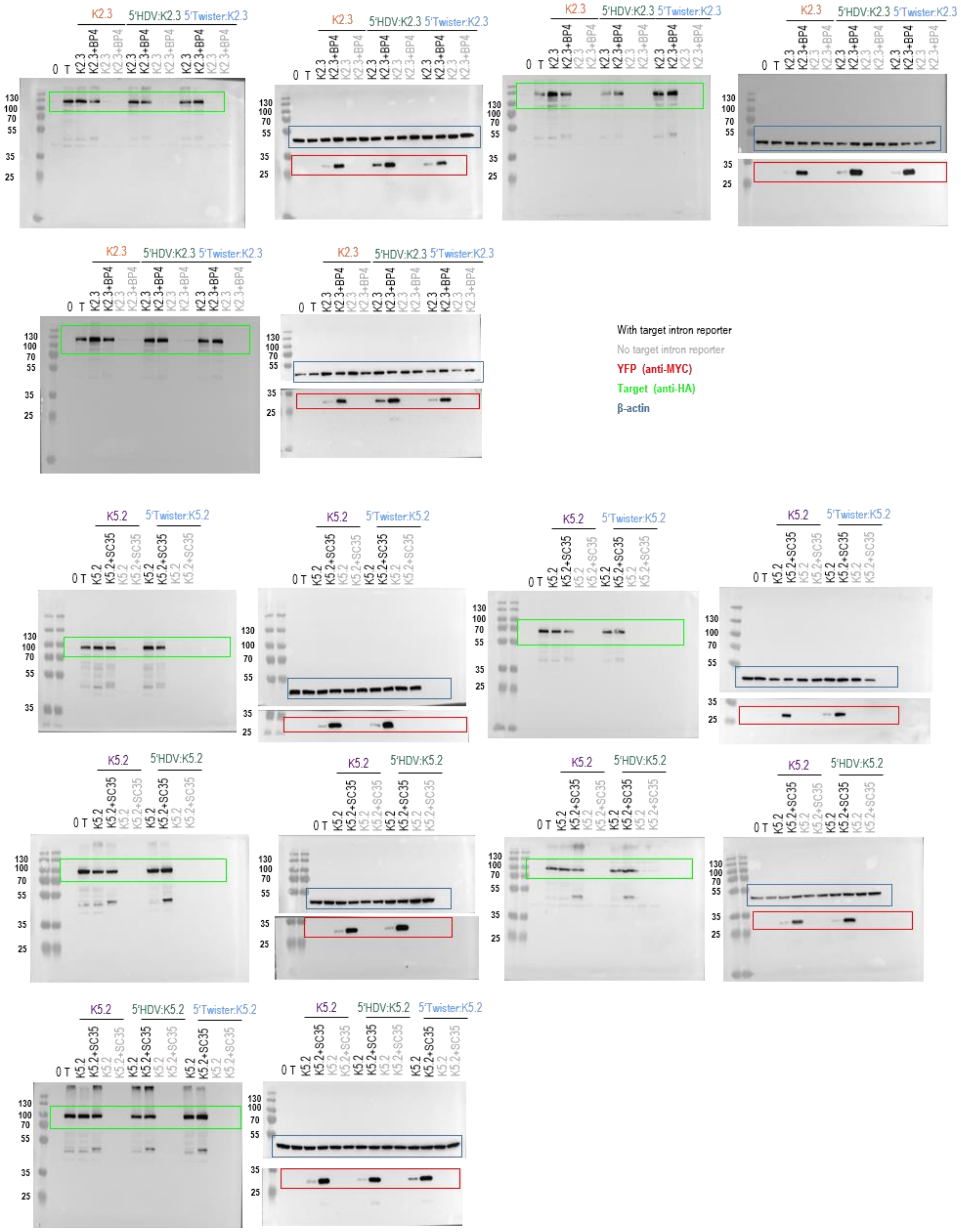

Figure S16: **Uncropped Western blots for generating Figure 6B and 6C**. The experiment was performed in three biological replicates. Cell lysates for the YFP (myc-tag) and β-actin were loaded on the same gel, while target intron reporter (HA-tag) was loaded on different gel. Composite images are presented to show the protein marker.

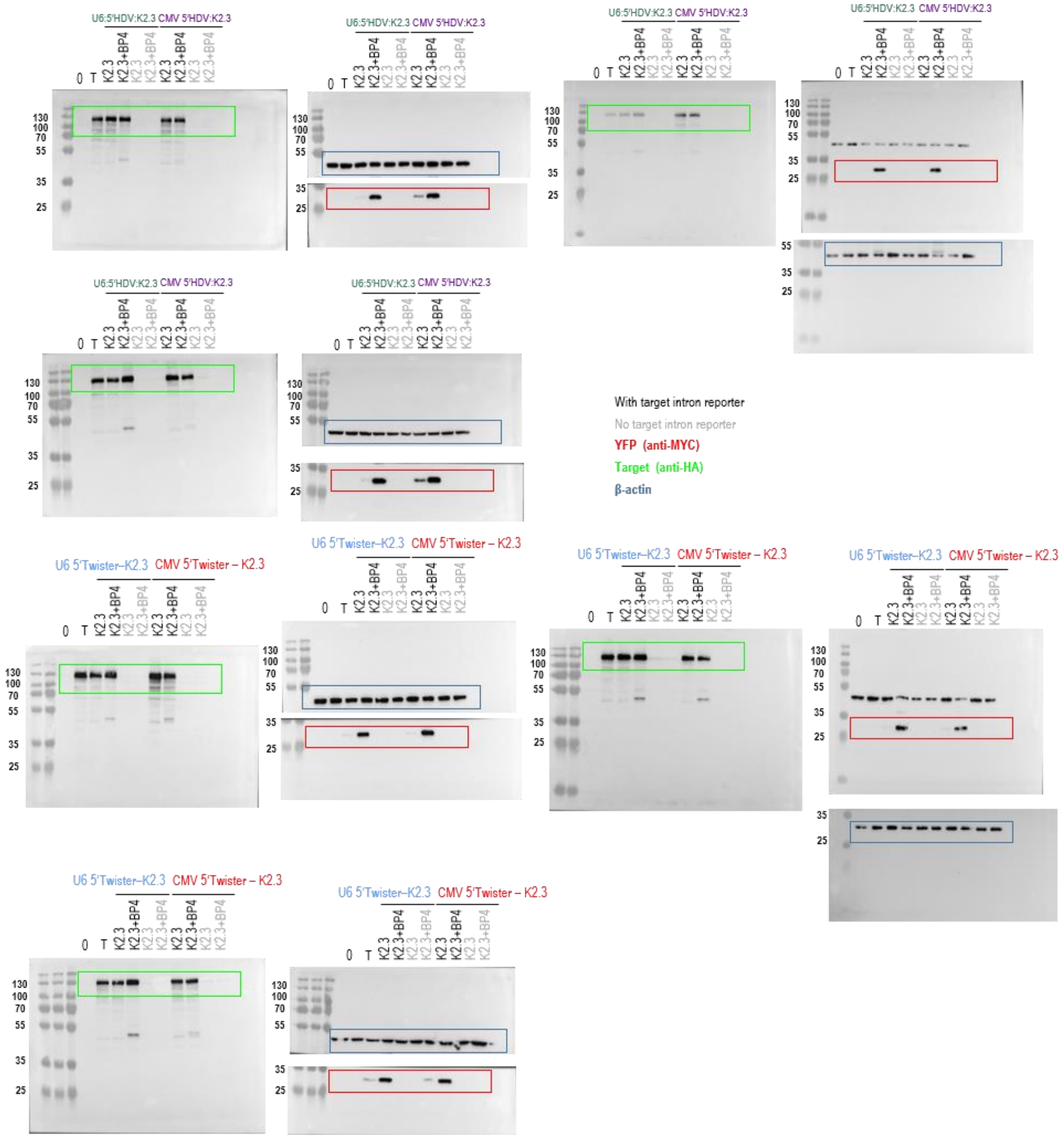

Figure S17: **Uncropped Western blots for generating Figure S10C and S10D**. The experiment was performed in three biological replicates. Cell lysates for the YFP (myc-tag) and β-actin were loaded on the same gel, while target intron reporter (HA-tag) was loaded on different gel. Composite images are presented to show the protein marker.

Table S1: **Computational prediction of splice sites strength with MaxEnt program.** MaxEnt program provides the entropy value for the 23 nucleotides of the 3' splice site (20 intronic+3exonic). Lower value indicates weaker splice site.

	CTNNB1 target intron				
	**Intron 2**	**Intron 3**	**Intron 5**	**Intron 6**	**PTM**
**3SS sequence**	atactgtttcgtatttatAGctg	gttgaattaaccttttccAGata	ggcttttttcttcttcccAGttc	atatatatatatctttctAGctc	ttttttccctttttttccAGgac
**MaxEt value**	7.67	4.66	8.58	7.07	12.39

Table S2: **A list and DNA sequences of constructs designed and tested for the screening of PTMs targeting introns 2, 3, 5 and 6.**

PTM ID	INTRON	Binding domain:spacer:intronic sequence (branch point, polypirimidine tract, 3 splice site):cYFP:linker:MycTag
**K.I2(rv)**	**2**	ctataaatacgaaacagtattagcattagtagattggaaatgttactttaatttaaaaaaaaagaatctgtgatatgacccattgaaatatttttagaaataaagtcaaatctgaaagacagccaagaaaagcagaatgatagccagggttagctcagtgatgaaatacctaatcccttagggaaccacctaacagttactcactgaatcagtggaagaatggtactgcatccaggctccagaagcagtcatccagactagattcctgctggtggcttgtttgctatttcaccaagccattaggaggagtgagcagaaaatggagcaaaaggtagcctgacaagtaagcagggagagaggaaagcagggggatctcagccagactggcttaatggcaacgaagcagagccccaattcagtaggactgacgtcactcagtcctccttactaactttttttcccttttttttccAGgacggcagcgtgcagctcgccgaccactaccagcagaacacccccatcggcgacggccccgtgctgctgcccgacaaccactacctgagctaccagtccgccctgagcaaagaccccaacgagaagcgcgatcacatggtcctgctggagttcgtgaccgccgccgggatcactctcggcatggacgagctgtacaaggggggtggaggctctgagcagaagctgatcagcgaggaggacctg
**K2.1**	**2**	tttcaccaagccattaggaggagtgagcagaaaatggagcaaaaggtagcctgacaagtaagcagggagagaggaaagcagggggatctcagccagactggcttaatggcaacgaagcagagccccaattcagtaactaaagatttaatgaggactgacgtcactcagtcctccttactaactttttttcccttttttttccAGgacggcagcgtgcagctcgccgaccactaccagcagaacacccccatcggcgacggccccgtgctgctgcccgacaaccactacctgagctaccagtccgccctgagcaaagaccccaacgagaagcgcgatcacatggtcctgctggagttcgtgaccgccgccgggatcactctcggcatggacgagctgtacaaggggggtggaggctctgagcagaagctgatcagcgaggaggacctg
**K2.2**	**2**	ctccagaagcagtcatccagactagattcctgctggtggcttgtttgctatttcaccaagccattaggaggagtgagcagaaaatggagcaaaaggtagcctgacaagtaagcagggagagaggaaagcagggggatctcagccagactgaggactgacgtcactcagtcctccttactaactttttttcccttttttttccAGgacggcagcgtgcagctcgccgaccactaccagcagaacacccccatcggcgacggccccgtgctgctgcccgacaaccactacctgagctaccagtccgccctgagcaaagaccccaacgagaagcgcgatcacatggtcctgctggagttcgtgaccgccgccgggatcactctcggcatggacgagctgtacaaggggggtggaggctctgagcagaagctgatcagcgaggaggacctg
**K2.3**	**2**	cacctaacagttactcactgaatcagtggaagaatggtactgcatccaggctccagaagcagtcatccagactagattcctgctggtggcttgtttgctatttcaccaagccattaggaggagtgagcagaaaatggagcaaaaggtagcaggactgacgtcactcagtcctccttactaactttttttcccttttttttccAGgacggcagcgtgcagctcgccgaccactaccagcagaacacccccatcggcgacggccccgtgctgctgcccgacaaccactacctgagctaccagtccgccctgagcaaagaccccaacgagaagcgcgatcacatggtcctgctggagttcgtgaccgccgccgggatcactctcggcatggacgagctgtacaaggggggtggaggctctgagcagaagctgatcagcgaggaggacctg
**K2.4**	**2**	atgatagccagggttagctcagtgatgaaatacctaatcccttagggaaccacctaacagttactcactgaatcagtggaagaatggtactgcatccaggctccagaagcagtcatccagactagattcctgctggtggcttgtttgctaaggactgacgtcactcagtcctccttactaactttttttcccttttttttccAGgacggcagcgtgcagctcgccgaccactaccagcagaacacccccatcggcgacggccccgtgctgctgcccgacaaccactacctgagctaccagtccgccctgagcaaagaccccaacgagaagcgcgatcacatggtcctgctggagttcgtgaccgccgccgggatcactctcggcatggacgagctgtacaaggggggtggaggctctgagcagaagctgatcagcgaggaggacctg
**K2.5**	**2**	aatatttttagaaataaagtcaaatctgaaagacagccaagaaaagcagaatgatagccagggttagctcagtgatgaaatacctaatcccttagggaaccacctaacagttactcactgaatcagtggaagaatggtactgcatccaggaggactgacgtcactcagtcctccttactaactttttttcccttttttttccAGgacggcagcgtgcagctcgccgaccactaccagcagaacacccccatcggcgacggccccgtgctgctgcccgacaaccactacctgagctaccagtccgccctgagcaaagaccccaacgagaagcgcgatcacatggtcctgctggagttcgtgaccgccgccgggatcactctcggcatggacgagctgtacaaggggggtggaggctctgagcagaagctgatcagcgaggaggacctg
**K2.6**	**2**	gaaatgttactttaatttaaaaaaaaagaatctgtgatatgacccattgaaatatttttagaaataaagtcaaatctgaaagacagccaagaaaagcagaatgatagccagggttagctcagtgatgaaatacctaatcccttagggaacaggactgacgtcactcagtcctccttactaactttttttcccttttttttccAGgacggcagcgtgcagctcgccgaccactaccagcagaacacccccatcggcgacggccccgtgctgctgcccgacaaccactacctgagctaccagtccgccctgagcaaagaccccaacgagaagcgcgatcacatggtcctgctggagttcgtgaccgccgccgggatcactctcggcatggacgagctgtacaaggggggtggaggctctgagcagaagctgatcagcgaggaggacctg
**K2.7**	**2**	ctccatcaaatcagctataaatacgaaacagtattagcattagtagattggaaatgttactttaatttaaaaaaaaagaatctgtgatatgacccattgaaatattttttagaaataaagtcaaatctgaaagacagccaagaaaagcagaaggactgacgtcactcagtcctccttactaactttttttcccttttttttccAGgacggcagcgtgcagctcgccgaccactaccagcagaacacccccatcggcgacggccccgtgctgctgcccgacaaccactacctgagctaccagtccgccctgagcaaagaccccaacgagaagcgcgatcacatggtcctgctggagttcgtgaccgccgccgggatcactctcggcatggacgagctgtacaaggggggtggaggctctgagcagaagctgatcagcgaggaggacctg
**K3.1**	**3**	agttcagcatttacctaagtatttgctatcctaaatggtaaaagtgacattgctattactctcttttcttcaccacaacattttatttaaactattatacactaacttttagttctcaaaactgcattctgactttcagtaaggcaatgaggactgacgtcactcagtcctccttactaactttttttcccttttttttccAGgacggcagcgtgcagctcgccgaccactaccagcagaacacccccatcggcgacggccccgtgctgctgcccgacaaccactacctgagctaccagtccgccctgagcaaagaccccaacgagaagcgcgatcacatggtcctgctggagttcgtgaccgccgccgggatcactctcggcatggacgagctgtacaaggggggtggaggctctgagcagaagctgatcagcgaggaggacctg
**K3.2**	**3**	atactgtccatcaatatctggaaaaggttaattcaacactcactatccacagttcagcatttacctaagtatttgctatcctaaatggtaaaagtgacattgctattactctcttttcttcaccacaacattttatttaaactattatacaggactgacgtcactcagtcctccttactaactttttttcccttttttttccAGgacggcagcgtgcagctcgccgaccactaccagcagaacacccccatcggcgacggccccgtgctgctgcccgacaaccactacctgagctaccagtccgccctgagcaaagaccccaacgagaagcgcgatcacatggtcctgctggagttcgtgaccgccgccgggatcactctcggcatggacgagctgtacaaggggggtggaggctctgagcagaagctgatcagcgaggaggacctg
**K5.1**	**5**	aatattgtgagtatactcttctactttttagcttcaagcattctgacatgaggactgacgtcactcagtcctccttactaactttttttcccttttttttccAGgacggcagcgtgcagctcgccgaccactaccagcagaacacccccatcggcgacggccccgtgctgctgcccgacaaccactacctgagctaccagtccgccctgagcaaagaccccaacgagaagcgcgatcacatggtcctgctggagttcgtgaccgccgccgggatcactctcggcatggacgagctgtacaaggggggtggaggctctgagcagaagctgatcagcgaggaggacctg
**K5.2**	**5**	tggtgaactgggaagaagaaaaaagcctcatcagaaatattgtgagtataaggactgacgtcactcagtcctccttactaactttttttcccttttttttccAGgacggcagcgtgcagctcgccgaccactaccagcagaacacccccatcggcgacggccccgtgctgctgcccgacaaccactacctgagctaccagtccgccctgagcaaagaccccaacgagaagcgcgatcacatggtcctgctggagttcgtgaccgccgccgggatcactctcggcatggacgagctgtacaaggggggtggaggctctgagcagaagctgatcagcgaggaggacctg
**K6.1**	**6**	gaatggtgtcatccagaaaccattcccacaacccattcatggaaaaactgttttccacgaaactggtccctggtgccaaaaaggttaggaacactgcatggaatgacatgacactggaggtgtccaatgctccatgaaaaccacataaagaggactgacgtcactcagtcctccttactaactttttttcccttttttttccAGgacggcagcgt

		gcagctcgccgaccactaccagcagaacacccccatcggcgacggccccgtgctgctgcccgacaaccactacctgagctaccagtccgccctgagcaaagacccccaacgagaagcgcgatcacatggtcctgctggagttcgtgaccgccgccgggatcactctcggcatggacgagctgtacaaggggggtggaggctctgagcagaagctgatcagcgaggaggacctg
**K6.2**	**6**	gagagtctcataggagtagaaaccctagtgtggactgcacatgcgagggatctaggctgcacgctccctatgagaatctaatgcctgattatctgaggtggaatggtgtcatccagaaaccattcccacaacccattcatggaaaaactgaggactgacgtcactcagtcctccttactaactttttttccctttttttccAGgacggcagcgtgcagctcgccgaccactaccagcagaacacccccatcggcgacggccccgtgctgctgcccgacaaccactacctgagctaccagtccgccctgagcaaagacccccaacgagaagcgcgatcacatggtcctgctggagttcgtgaccgccgccgggatcactctcggcatggacgagctgtacaaggggggtggaggctctgagcagaagctgatcagcgaggaggacctg
**K6.3**	**6**	ccactagccagtatgatgagctagaaagatatatatatatatcttgttagtcaaggaatcttgatcacatgtctgagaagagccatattaccaacctattttattatacaagaatttcagccagcttgccattggacctaaatgtattccaggactgacgtcactcagtcctccttactaactttttttccctttttttccAGgacggcagcgtgcagctcgccgaccactaccagcagaacacccccatcggcgacggccccgtgctgctgcccgacaaccactacctgagctaccagtccgccctgagcaaagaccccaacgagaagcgcgatcacatggtcctgctggagttcgtgaccgccgccgggatcactctcggcatggacgagctgtacaaggggggtggaggctctgagcagaagctgatcagcgaggaggacctg
**K6.4**	**6**	ttattatacaagaatttcagccagcttgccattggacctaaatgtattcctggaccccatcactcagacccccatactggggccctatccaacttgtccttttaaagcattccactcaaattcagacatcccagtcctccaaacaatgtaaggactgacgtcactcagtcctccttactaactttttttccctttttttccAGgacggcagcgtgcagctcgccgaccactaccagcagaacacccccatcggcgacggccccgtgctgctgcccgacaaccactacctgagctaccagtccgccctgagcaaagaccccaacgagaagcgcgatcacatggtcctgctggagttcgtgaccgccgccgggatcactctcggcatggacgagctgtacaaggggggtggaggctctgagcagaagctgatcagcgaggaggacctg
**K6.5**	**6**	aagaacagaaatgaactgggctgcacagcaggaggtaagtggcaggtgagcgagcattacctggcttgagctctacctcctgtcagatcaactgcaccatgagagtctcataggagtagaaaccctagtgtggactgcacatgcgagggaaggactgacgtcactcagtcctccttactaactttttttccctttttttccAGgacggcagcgtgcagctcgccgaccactaccagcagaacacccccatcggcgacggccccgtgctgctgcccgacaaccactacctgagctaccagtccgccctgagcaaagacccccaacgagaagcgcgatcacatggtcctgctggagttcgtgaccgccgccgggatcactctcggcatggacgagctgtacaaggggggtggaggctctgagcagaagctgatcagcgaggaggacctg
**K6.6**	**6**	atctacatcccacctaagaacaaaagtgtgaagagaagcttgcctaccatatccctactacttcgcatagtgctttacatatggaaactcaaaaatttaaaagaacagaaatgaactgggctgcacagcaggaggtaagtggcaggtgagaggactgacgtcactcagtcctccttactaactttttttccctttttttccAGgacggcagcgtgcagctcgccgaccactaccagcagaacacccccatcggcgacggccccgtgctgctgcccgacaaccactacctgagctaccagtccgccctgagcaaagacccccaacgagaagcgcgatcacatggtcctgctggagttcgtgaccgccgccgggatcactctcggcatggacgagctgtacaaggggggtggaggctctgagcagaagctgatcagcgaggaggacctg
**K6.7**	**6**	ttgaaccctcaaaaagagtcagtttcaacttgtgttatgtgccctcatagccccacttcttcccactacacaaattaaatattaggttattattcccaacatctacatcccacctaagaacaaaagtgtgaagagaagcttgcctaccataggactgacgtcactcagtcctccttactaactttttttccctttttttccAGgacggcagcgtgcagctcgccgaccactaccagcagaacacccccatcggcgacggccccgtgctgctgcccgacaaccactacctgagctaccagtccgccctgagcaaagaccccaacgagaagcgcgatcacatggtcctgctggagttcgtgaccgccgccgggatcactctcggcatggacgagctgtacaaggggggtggaggctctgagcagaagctgatcagcgaggaggacctg
**K6.8**	**6**	tcgtttcaaagatgttgttttctcacctaaccttcacctagtgaatcccttctcatccttgaacactgaactcaactatcacttcctccaagaggtctccttgaaccctcaaaaagagtcagtttcaacttgtgttatgtgccctcatagaggactgacgtcactcagtcctccttactaactttttttccctttttttccAGgacggcagcgtgcagctcgccgaccactaccagcagaacacccccatcggcgacggccccgtgctgctgcccgacaaccactacctgagctaccagtccgccctgagcaaagaccccaacgagaagcgcgatcacatggtcctgctggagttcgtgaccgccgccgggatcactctcggcatggacgagctgtacaaggggggtggaggctctgagcagaagctgatcagcgaggaggacctg
**K6.9**	**6**	atgagatctgctaagtctatctcaatagcctcacctcaaacctttcttcctccttactattagtatttcttaaattcccaaaactttccatctccttccttcgtttcaaagatgttgttttctcacctaaccttcacctagtgaatccctaggactgacgtcactcagtcctccttactaactttttttccctttttttccAGgacggcagcgtgcagctcgccgaccactaccagcagaacacccccatcggcgacggccccgtgctgctgcccgacaaccactacctgagctaccagtccgccctgagcaaagaccccaacgagaagcgcgatcacatggtcctgctggagttcgtgaccgccgccgggatcactctcggcatggacgagctgtacaaggggggtggaggctctgagcagaagctgatcagcgaggaggacctg
**K6.10**	**6**	ccctttcaacactgccccaattttctaaaaacaaatttcatcatttcaggcttctgctgaaatttcccattgtgcttttaaacatgctctacaaagccctatgagatctgctaagtctatctcaatagcctcacctcaaacctttcttccaaggactgacgtcactcagtcctccttactaactttttttccctttttttccAGgacggcagcgtgcagctcgccgaccactaccagcagaacacccccatcggcgacggccccgtgctgctgcccgacaaccactacctgagctaccagtccgccctgagcaaagaccccaacgagaagcgcgatcacatggtcctgctggagttcgtgaccgccgccgggatcactctcggcatggacgagctgtacaaggggggtggaggctctgagcagaagctgatcagcgaggaggacctg
**K6.11**	**6**	ctcatcatttggccctttaattttcctaattatatccaccttaaaaacagataccataaaatgatcattcatgcttgtcctttcattccctgtatatcttccctttcaacactgccccaattttctaaaaacaaatttcatcatttcaggaggactgacgtcactcagtcctccttactaactttttttccctttttttccAGgacggcagcgtgcagctcgccgaccactaccagcagaacacccccatcggcgacggccccgtgctgctgcccgacaaccactacctgagctaccagtccgccctgagcaaagaccccaacgagaagcgcgatcacatggtcctgctggagttcgtgaccgccgccgggatcactctcggcatggacgagctgtacaaggggggtggaggctctgagcagaagctgatcagcgaggaggacctg
**K6.12**	**6**	aatttagaaactatgttggtaggcaagagtaatgccctaagatgaaagttatatcaaaactgattcactgaaaatttgaaccagaactggcacttaactcctcatcatttggccctttaattttcctaattatatccaccttaaaaacagaggactgacgtcactcagtcctccttactaactttttttccctttttttccAGgacggcagcgtgcagctcgccgaccactaccagcagaacacccccatcggcgacggccccgtgctgctgcccgacaaccactacctgagctaccagtccgccctgagcaaagaccccaacgagaagcgcgatcacatggtcctgctggagttcgtgaccgccgccgggatcactctcggcatggacgagctgtacaaggggggtggaggctctgagcagaagctgatcagcgaggaggacctg
**K6.13**	**6**	ttaaagcattccactcaaattcagacatcccagtcctccaaacaatgtatcagtttgggcccaatttccttcttcccacagtgatcacaccaaaagaaaaaaatttagaaactatgttggtaggcaagagtaatgccctaagatgaaagtaggactgacgtcactcagtcctccttactaactttttttccctttttttccAGgacggcagcgtgcagctcgccgaccactaccagcagaacacccccatcggcgacggccccgtgctgctgcccgacaaccactacctgagctaccagtccgccctgagcaaagacccccaacgagaagcgcgatcacatggtcctgctggagttcgtgaccgccgccgggatcactctcggcatggacgagctgtacaaggggggtggaggctctgagcagaagctgatcagcgaggaggacctg

Table S3. **List and DNA sequences of constructs designed and tested to identify the most effective PTMs (K2.3, K5.2, and K6.5) in combination with ribozymes.**

PTM ID	Intron	5'Ribozyme:SPACER:binding domain: intronic sequence(spacer:branch point:polypirimidine tract:3' splice site)cYFP:linker:MycTag: 3'Ribozyme
**5'Twister:K2.3**	**2**	ccgcctaacactgccaatgccggtcccaagcccggataaaaagtggagggggcggcacctaacagttactcactgaatcagtggaagaatggtactgcatccaggctccagaagcagtcatccagactagattcctgctggtggcttgtttgctatttcaccaagccattaggaggagtgagcagaaaatggagcaaaaggtagcaggactgacgtcactcagtcctccttactaactttttttcccttttttttccAGgacggcagcgtgcagctcgccgaccactaccagcagaacacccccatcggcgacggccccgtgctgctgcccgacaaccactacctgagctaccagtccgccctgagcaaagaccccaacgagaagcgcgatcacatggtcctgctggagttcgtgaccgccgccgggatcactctcggcatggacgagctgtacaaggggggtggaggctctgagcagaagctgatcagcgaggaggacctg
**5'Twister:K2.3 MUT**	**2**	ccgcctaactcttccaatgccggtcccaagcccggataaaaagtggagggggcggcacctaacagttactcactgaatcagtggaagaatggtactgcatccaggctccagaagcagtcatccagactagattcctgctggtggcttgtttgctatttcaccaagccattaggaggagtgagcagaaaatggagcaaaaggtagcaggactgacgtcactcagtcctccttactaactttttttcccttttttttccAGgacggcagcgtgcagctcgccgaccactaccagcagaacacccccatcggcgacggccccgtgctgctgcccgacaaccactacctgagctaccagtccgccctgagcaaagaccccaacgagaagcgcgatcacatggtcctgctggagttcgtgaccgccgccgggatcactctcggcatggacgagctgtacaaggggggtggaggctctgagcagaagctgatcagcgaggaggacctg
**5'HDV:K2.3**	**2**	ggccggcatggtcccagcctcctcgctggcgccggctgggcaacatgcttcggcatggcgaatgggacacacctaacagttactcactgaatcagtggaagaatggtactgcatccaggctccagaagcagtcatccagactagattcctgctggtggcttgtttgctatttcaccaagccattaggaggagtgagcagaaaatggagcaaaaggtagcaggactgacgtcactcagtcctccttactaactttttttcccttttttttccAGgacggcagcgtgcagctcgccgaccactaccagcagaacacccccatcggcgacggccccgtgctgctgcccgacaaccactacctgagctaccagtccgccctgagcaaagaccccaacgagaagcgcgatcacatggtcctgctggagttcgtgaccgccgccgggatcactctcggcatggacgagctgtacaaggggggtggaggctctgagcagaagctgatcagcgaggaggacctg
**5'HDV:K2.3 MUT**	**2**	ggccggcatggtcccagcctcctcgctggcgccggctgggcaacatgcttcggcatggagaatgggacacacctaacagttactcactgaatcagtggaagaatggtactgcatccaggctccagaagcagtcatccagactagattcctgctggtggcttgtttgctatttcaccaagccattaggaggagtgagcagaaaatggagcaaaaggtagcaggactgacgtcactcagtcctccttactaactttttttcccttttttttccAGgacggcagcgtgcagctcgccgaccactaccagcagaacacccccatcggcgacggccccgtgctgctgcccgacaaccactacctgagctaccagtccgccctgagcaaagaccccaacgagaagcgcgatcacatggtcctgctggagttcgtgaccgccgccgggatcactctcggcatggacgagctgtacaaggggggtggaggctctgagcagaagctgatcagcgaggaggacctg
**5'HH:K2.3**	**2**	taggtgctgatgagtccgtgaggacgaaacgagtaagctcgtccacctaacagttactcactgaatcagtggaagaatggtactgcatccaggctccagaagcagtcatccagactagattcctgctggtggcttgtttgctatttcaccaagccattaggaggagtgagcagaaaatggagcaaaaggtagcaggactgacgtcactcagtcctccttactaactttttttcccttttttttccAGgacggcagcgtgcagctcgccgaccactaccagcagaacacccccatcggcgacggccccgtgctgctgcccgacaaccactacctgagctaccagtccgccctgagcaaagaccccaacgagaagcgcgatcacatggtcctgctggagttcgtgaccgccgccgggatcactctcggcatggacgagctgtacaaggggggtggaggctctgagcagaagctgatcagcgaggaggacctg
**3'Twister:K2.3**	**2**	cacctaacagttactcactgaatcagtggaagaatggtactgcatccaggctccagaagcagtcatccagactagattcctgctggtggcttgtttgctatttcaccaagccattaggaggagtgagcagaaaatggagcaaaaggtagcaggactgacgtcactcagtcctccttactaactttttttcccttttttttccAGgacggcagcgtgcagctcgccgaccactaccagcagaacacccccatcggcgacggccccgtgctgctgcccgacaaccactacctgagctaccagtccgccctgagcaaagaccccaacgagaagcgcgatcacatggtcctgctggagttcgtgaccgccgccgggatcactctcggcatggacgagctgtacaaggggggtggaggctctgagcagaagctgatcagcgaggaggacctgccgcctaacactgccaatgccggtcccaagcccggataaaaagtggagggggcgg
**3'HDV:K2.3**	**2**	cacctaacagttactcactgaatcagtggaagaatggtactgcatccaggctccagaagcagtcatccagactagattcctgctggtggcttgtttgctatttcaccaagccattaggaggagtgagcagaaaatggagcaaaaggtagcaggactgacgtcactcagtcctccttactaactttttttcccttttttttccAGgacggcagcgtgcagctcgccgaccactaccagcagaacacccccatcggcgacggccccgtgctgctgcccgacaaccactacctgagctaccagtccgccctgagcaaagaccccaacgagaagcgcgatcacatggtcctgctggagttcgtgaccgccgccgggatcactctcggcatggacgagctgtacaaggggggtggaggctctgagcagaagctgatcagcgaggaggacctgggccggcatggtcccagcctcctcgctggcgccggctgggcaacatgcttcggcatggcgaatgggac
**5'Twister:K5.2**	**5**	ccgcctaacactgccaatgccggtcccaagcccggataaaaagtggagggggcggtggtgaactgggaagaagaaaaaagcctcatcagaaatattgtgagtataaggactgacgtcactcagtcctccttactaactttttttcccttttttttccAGgacggcagcgtgcagctcgccgaccactaccagcagaacacccccatcggcgacggccccgtgctgctgcccgacaaccactacctgagctaccagtccgccctgagcaaagaccccaacgagaagcgcgatcacatggtcctgctggagttcgtgaccgccgccgggatcactctcggcatggacgagctgtacaaggggggtggaggctctgagcagaagctgatcagcgaggaggacctg
**5'HDV:K5.2**	**5**	ggccggcatggtcccagcctcctcgctggcgccggctgggcaacatgcttcggcatggcgaatgggactggtgaactgggaagaagaaaaaagcctcatcagaaatattgtgagtataaggactgacgtcactcagtcctccttactaactttttttcccttttttttccAGgacggcagcgtgcagctcgccgaccactaccagcagaacacccccatcggcgacggccccgtgctgctgcccgacaaccactacctgagctaccagtccgccctgagcaaagaccccaacgagaagcgcgatcacatggtcctgctggagttcgtgaccgccgccgggatcactctcggcatggacgagctgtacaaggggggtggaggctctgagcagaagctgatcagcgaggaggacctg
**5'HH:K5.2**	**5**	tcaccactgatgagtccgtgaggacgaaacgagtaagctcgtcggtgaactgggaagaagaaaaaagcctcatcagaaatattgtgagtataaggactgacgtcactcagtcctccttactaactttttttcccttttttttccAGgacggcagcgtgcagctcgccgaccactaccagcagaacacccccatcggcgacggccccgtgctgctgcccgacaaccactacctgagctaccagtccgccctgagcaaagaccccaacgagaagcgcgatcacatggtcctgctggagttcgtgaccgccgccgggatcactctcggcatggacgagctgtacaaggggggtggaggctctgagcagaagctgatcagcgaggaggacctg
**3'Twister:K5.2**	**5**	tggtgaactgggaagaagaaaaaagcctcatcagaaatattgtgagtataaggactgacgtcactcagtcctccttactaactttttttcccttttttttccAGgacggcagcgtgcagctcgccgaccactaccagcagaacacccccatcggcgacggccccgtgctgctgcccgacaaccactacctgagctaccagtccgccctgagcaaagaccccaacgagaagcgcgatcacatggtcctgctggagttcgtgaccgccgccgggatcactctcggcatggacgagctgtacaaggggggtggaggctctgagcagaagctgatcagcgaggaggacctg ccgcctaacactgccaatgccggtcccaagcccggataaaaagtggagggggcgg

**3'HDV:K5.2**	**5**	tggtgaactgggaagaagaaaaaagcctcatcagaaatattgtgagtataaggactgacgtcactcagtcctccttactaactttttttcccttttttttccAGgacggcagcgtgcagctcgccgaccactaccagcagaacacccccatcggcgacggccccgtgctgctgcccgacaaccactacctgagctaccagtccgccctgagcaaagaccccaacgagaagcgcgatcacatggtcctgctggagttcgtgaccgccgccgggatcactctcggcatggacgagctgtacaagggggtggaggctctgagcagaagctgatcagcgaggaggacctgggccggcatggtcccagcctcctcgctggcgccggctgggcaacatgcttcggcatggcgaatgggac
**5'Twister:K6.5**	**6**	ccgcctaacactgccaatgccggtcccaagcccggataaaaagtggagggggcggaagaacagaaatgaactgggctgcacagcaggaggtaagtggcaggtgagcgagcattacctggcttgagctctacctcctgtcagatcaactgcaccatgagagtctcataggagtagaaaccctagtgtggactgcacatgcgagggaaggactgacgtcactcagtcctccttactaactttttttcccttttttttccAGgacggcagcgtgcagctcgccgaccactaccagcagaacacccccatcggcgacggccccgtgctgctgcccgacaaccactacctgagctaccagtccgccctgagcaaagaccccaacgagaagcgcgatcacatggtcctgctggagttcgtgaccgccgccgggatcactctcggcatggacgagctgtacaagggggtggaggctctgagcagaagctgatcagcgaggaggacctg
**5'HDV:K6.5**	**6**	ggccggcatggtcccagcctcctcgctggcgccggctgggcaacatgcttcggcatggcgaatgggacaagaacagaaatgaactgggctgcacagcaggaggtaagtggcaggtgagcgagcattacctggcttgagctctacctcctgtcagatcaactgcaccatgagagtctcataggagtagaaaccctagtgtggactgcacatgcgagggaaggactgacgtcactcagtcctccttactaactttttttcccttttttttccAGgacggcagcgtgcagctcgccgaccactaccagcagaacacccccatcggcgacggccccgtgctgctgcccgacaaccactacctgagctaccagtccgccctgagcaaagaccccaacgagaagcgcgatcacatggtcctgctggagttcgtgaccgccgccgggatcactctcggcatggacgagctgtacaagggggtggaggctctgagcagaagctgatcagcgaggaggacctg
**5'HH:K6.5**	**6**	gttcttctgatgagtccgtgaggacgaaacgagtaagctcgtcaagaacagaaatgaactgggctgcacagcaggaggtaagtggcaggtgagcgagcattacctggcttgagctctacctcctgtcagatcaactgcaccatgagagtctcataggagtagaaaccctagtgtggactgcacatgcgagggaaggactgacgtcactcagtcctccttactaactttttttcccttttttttccAGgacggcagcgtgcagctcgccgaccactaccagcagaacacccccatcggcgacggccccgtgctgctgcccgacaaccactacctgagctaccagtccgccctgagcaaagaccccaacgagaagcgcgatcacatggtcctgctggagttcgtgaccgccgccgggatcactctcggcatggacgagctgtacaagggggtggaggctctgagcagaagctgatcagcgaggaggacctg
**3'Twister:K6.5**	**6**	aagaacagaaatgaactgggctgcacagcaggaggtaagtggcaggtgagcgagcattacctggcttgagctctacctcctgtcagatcaactgcaccatgagagtctcataggagtagaaaccctagtgtggactgcacatgcgagggaaggactgacgtcactcagtcctccttactaactttttttcccttttttttccAGgacggcagcgtgcagctcgccgaccactaccagcagaacacccccatcggcgacggccccgtgctgctgcccgacaaccactacctgagctaccagtccgccctgagcaaagaccccaacgagaagcgcgatcacatggtcctgctggagttcgtgaccgccgccgggatcactctcggcatggacgagctgtacaagggggtggaggctctgagcagaagctgatcagcgaggaggacctg ccgcctaacactgccaatgccggtcccaagcccggataaaaagtggagggggcgg
**3'HDV:K6.5**	**6**	aagaacagaaatgaactgggctgcacagcaggaggtaagtggcaggtgagcgagcattacctggcttgagctctacctcctgtcagatcaactgcaccatgagagtctcataggagtagaaaccctagtgtggactgcacatgcgagggaaggactgacgtcactcagtcctccttactaactttttttcccttttttttccAGgacggcagcgtgcagctcgccgaccactaccagcagaacacccccatcggcgacggccccgtgctgctgcccgacaaccactacctgagctaccagtccgccctgagcaaagaccccaacgagaagcgcgatcacatggtcctgctggagttcgtgaccgccgccgggatcactctcggcatggacgagctgtacaagggggtggaggctctgagcagaagctgatcagcgaggaggacctgggccggcatggtcccagcctcctcgctggcgccggctgggcaacatgcttcggcatggcgaatgggac

Table S4: **A list and DNA sequences of constructs designed and tested to optimize BD length for PTMs K2.3 and K6.5.**

PTM ID	Intron	binding domain: intronic sequence(spacer:branch point:polypirimidine tract:3' splice site)cYFP:linker:MycTag
**K2.3(100nt.1)**	**2**	gtggaagaatggtactgcatccaggctccagaagcagtcatccagactagattcctgctggtggcttgtttgctatttcaccaagccattaggaggagtgagg<br>actgacgtcactcagtcctcctttactaactttttttcccttttttttccAGgacggcagcgtgcagctcgccgaccactaccagcagaacacccccatcggcgac<br>ggccccgtgctgctgcccgacaaccactacctgagctaccagtccgccctgagcaaagaccccaacgagaagcgcgatcacatggtcctgctggagttcgtg<br>accgccgccgggatcactctcggcatggacgagctgtacaaggggggtggaggctctgagcagaagctgatcagcgaggaggacctg
**K2.3(100nt.2)**	**2**	ctccagaagcagtcatccagactagattcctgctggtggcttgtttgctatttcaccaagccattaggaggagtgagcagaaaatggagcaaaaggtagcag<br>gactgacgtcactcagtcctcctttactaactttttttcccttttttttccAGgacggcagcgtgcagctcgccgaccactaccagcagaacacccccatcggcga<br>cggccccgtgctgctgcccgacaaccactacctgagctaccagtccgccctgagcaaagaccccaacgagaagcgcgatcacatggtcctgctggagttcgt<br>gaccgccgccgggatcactctcggcatggacgagctgtacaaggggggtggaggctctgagcagaagctgatcagcgaggaggacctg
**K2.3(100nt.3)**	**2**	cacctaacagttactcactgaatcagtggaagaatggtactgcatccaggctccagaagcagtcatccagactagattcctgctggtggcttgtttgctaagg<br>actgacgtcactcagtcctcctttactaactttttttcccttttttttccAGgacggcagcgtgcagctcgccgaccactaccagcagaacacccccatcggcgac<br>ggccccgtgctgctgcccgacaaccactacctgagctaccagtccgccctgagcaaagaccccaacgagaagcgcgatcacatggtcctgctggagttcgtg<br>accgccgccgggatcactctcggcatggacgagctgtacaaggggggtggaggctctgagcagaagctgatcagcgaggaggacctg
**K2.3(50nt.1)**	**2**	ctccagaagcagtcatccagactagattcctgctggtggcttgtttgctaaggactgacgtcactcagtcctcctttactaactttttttcccttttttttccAGgac<br>ggcagcgtgcagctcgccgaccactaccagcagaacacccccatcggcgacggccccgtgctgctgcccgacaaccactacctgagctaccagtccgccct<br>gagcaaagaccccaacgagaagcgcgatcacatggtcctgctggagttcgtgaccgccgccgggatcactctcggcatggacgagctgtacaaggggggt<br>ggaggctctgagcagaagctgatcagcgaggaggacctg
**K2.3(50nt.2)**	**2**	tttcaccaagccattaggaggagtgagcagaaaatggagcaaaaggtagcaggactgacgtcactcagtcctcctttactaactttttttcccttttttttccAGg<br>acggcagcgtgcagctcgccgaccactaccagcagaacacccccatcggcgacggccccgtgctgctgcccgacaaccactacctgagctaccagtccgcc<br>ctgagcaaagaccccaacgagaagcgcgatcacatggtcctgctggagttcgtgaccgccgccgggatcactctcggcatggacgagctgtacaaggggg<br>gtggaggctctgagcagaagctgatcagcgaggaggacctg
**K2.3(50nt.3)**	**2**	cacctaacagttactcactgaatcagtggaagaatggtactgcatccaggaggactgacgtcactcagtcctcctttactaactttttttcccttttttttccAGga<br>cggcagcgtgcagctcgccgaccactaccagcagaacacccccatcggcgacggccccgtgctgctgcccgacaaccactacctgagctaccagtccgccc<br>tgagcaaagaccccaacgagaagcgcgatcacatggtcctgctggagttcgtgaccgccgccgggatcactctcggcatggacgagctgtacaaggggggt<br>ggaggctctgagcagaagctgatcagcgaggaggacctg
**K6.5(100nt.1)**	**6**	cagcaggaggtaagtggcaggtgagcgagcattacctggcttgagctctacctcctgtcagatcaactgcaccatgagagtctcataggagtagaaaccca<br>ggactgacgtcactcagtcctcctttactaactttttttcccttttttttccAGgacggcagcgtgcagctcgccgaccactaccagcagaacacccccatcggcg<br>acggccccgtgctgctgcccgacaaccactacctgagctaccagtccgccctgagcaaagaccccaacgagaagcgcgatcacatggtcctgctggagttc<br>gtgaccgccgccgggatcactctcggcatggacgagctgtacaaggggggtggaggctctgagcagaagctgatcagcgaggaggacctg
**K6.5(100nt.2)**	**6**	cgagcattacctggcttgagctctacctcctgtcagatcaactgcaccatgagagtctcataggagtagaaaccctagtgtggactgcacatgcgagggaag<br>gactgacgtcactcagtcctcctttactaactttttttcccttttttttccAGgacggcagcgtgcagctcgccgaccactaccagcagaacacccccatcggcga<br>cggccccgtgctgctgcccgacaaccactacctgagctaccagtccgccctgagcaaagaccccaacgagaagcgcgatcacatggtcctgctggagttcgt<br>gaccgccgccgggatcactctcggcatggacgagctgtacaaggggggtggaggctctgagcagaagctgatcagcgaggaggacctg
**K6.5(100nt.3)**	**6**	aagaacagaaatgaactgggctgcacagcaggaggtaagtggcaggtgagcgagcattacctggcttgagctctacctcctgtcagatcaactgcaccata<br>ggactgacgtcactcagtcctcctttactaactttttttcccttttttttccAGgacggcagcgtgcagctcgccgaccactaccagcagaacacccccatcggcg<br>acggccccgtgctgctgcccgacaaccactacctgagctaccagtccgccctgagcaaagaccccaacgagaagcgcgatcacatggtcctgctggagttc<br>gtgaccgccgccgggatcactctcggcatggacgagctgtacaaggggggtggaggctctgagcagaagctgatcagcgaggaggacctg
**K6.5(50nt.1)**	**6**	cgagcattacctggcttgagctctacctcctgtcagatcaactgcaccataggactgacgtcactcagtcctcctttactaactttttttcccttttttttccAGgac<br>ggcagcgtgcagctcgccgaccactaccagcagaacacccccatcggcgacggccccgtgctgctgcccgacaaccactacctgagctaccagtccgccct<br>gagcaaagaccccaacgagaagcgcgatcacatggtcctgctggagttcgtgaccgccgccgggatcactctcggcatggacgagctgtacaaggggggt<br>ggaggctctgagcagaagctgatcagcgaggaggacctg
**K6.5(50nt.2)**	**6**	gagagtctcataggagtagaaaccctagtgtggactgcacatgcgagggaaggactgacgtcactcagtcctcctttactaactttttttcccttttttttccAGg<br>acggcagcgtgcagctcgccgaccactaccagcagaacacccccatcggcgacggccccgtgctgctgcccgacaaccactacctgagctaccagtccgcc<br>ctgagcaaagaccccaacgagaagcgcgatcacatggtcctgctggagttcgtgaccgccgccgggatcactctcggcatggacgagctgtacaaggggg<br>gtggaggctctgagcagaagctgatcagcgaggaggacctg
**K6.5(50nt.3)**	**6**	aagaacagaaatgaactgggctgcacagcaggaggtaagtggcaggtgagaggactgacgtcactcagtcctcctttactaactttttttcccttttttttccAG<br>gacggcagcgtgcagctcgccgaccactaccagcagaacacccccatcggcgacggccccgtgctgctgcccgacaaccactacctgagctaccagtccgc<br>cctgagcaaagaccccaacgagaagcgcgatcacatggtcctgctggagttcgtgaccgccgccgggatcactctcggcatggacgagctgtacaaggggg<br>gtggaggctctgagcagaagctgatcagcgaggaggacctg

Table S5: **A list and corresponding DNA sequences of constructs designed and tested for combined PTM candidates K2.3 and K6.5.**

	Intron	asRNA-spacer-binding domain: intronic sequence(spacer:branch point:polypirimidine tract:3' splice site)cYFP:linker:MycTag
**BP4:K1.2**	**2**	gccatgtccaactccatcaaatcagctatacacctaacagttactcactgaatcagtggaagaatggtactgcatccaggctccagaagcagtcatccagactagattcctgctggtggcttgtttgctatttcaccaagccattaggaggagtgagcagaaaatggagcaaaaggtagcaggactgacgtcactcagtcctccttactaactttttttccctttttttccAGgacggcagcgtgcagctcgccgaccactaccagcagaacacccccatcggcgacggccccgtgctgctgcccgacaaccactacctgagctaccagtccgccctgagcaaagaccccaacgagaagcgcgatcacatggtcctgctggagttcgtgaccgccgccgggatcactctcggcatggacgagctgtacaaggggggtggaggctctgagcagaagctgatcagcgaggaggacctg
**BP4:CTA:K1.2**	**2**	gccatgtccaactccatcaaatcagctataCTAcacctaacagttactcactgaatcagtggaagaatggtactgcatccaggctccagaagcagtcatccagactagattcctgctggtggcttgtttgctatttcaccaagccattaggaggagtgagcagaaaatggagcaaaaggtagcaggactgacgtcactcagtcctccttactaactttttttccctttttttccAGgacggcagcgtgcagctcgccgaccactaccagcagaacacccccatcggcgacggccccgtgctgctgcccgacaaccactacctgagctaccagtccgccctgagcaaagaccccaacgagaagcgcgatcacatggtcctgctggagttcgtgaccgccgccgggatcactctcggcatggacgagctgtacaaggggggtggaggctctgagcagaagctgatcagcgaggaggacctg
**BP4:CTT:K1.2**	**2**	gccatgtccaactccatcaaatcagctataCTTcacctaacagttactcactgaatcagtggaagaatggtactgcatccaggctccagaagcagtcatccagactagattcctgctggtggcttgtttgctatttcaccaagccattaggaggagtgagcagaaaatggagcaaaaggtagcaggactgacgtcactcagtcctccttactaactttttttccctttttttccAGgacggcagcgtgcagctcgccgaccactaccagcagaacacccccatcggcgacggccccgtgctgctgcccgacaaccactacctgagctaccagtccgccctgagcaaagaccccaacgagaagcgcgatcacatggtcctgctggagttcgtgaccgccgccgggatcactctcggcatggacgagctgtacaaggggggtggaggctctgagcagaagctgatcagcgaggaggacctg
**SF2/ASF:K6.5**	**6**	ccacagtagtttttcgtaagtataggtcctaagaacagaaatgaactgggctgcacagcaggaggtaagtggcaggtgagcgagcattacctggcttgagctctacctcctgtcagatcaactgcaccatgagagtctcataggagtagaaaccctagtgtggactgcacatgcgagggaaggactgacgtcactcagtcctccttactaactttttttccctttttttccAGgacggcagcgtgcagctcgccgaccactaccagcagaacacccccatcggcgacggccccgtgctgctgcccgacaaccactacctgagctaccagtccgccctgagcaaagaccccaacgagaagcgcgatcacatggtcctgctggagttcgtgaccgccgccgggatcactctcggcatggacgagctgtacaaggggggtggaggctctgagcagaagctgatcagcgaggaggacctg
**SF2/ASF:CGC: K6.5**	**6**	ccacagtagtttttcgtaagtataggtcctCGCaagaacagaaatgaactgggctgcacagcaggaggtaagtggcaggtgagcgagcattacctggcttgagctctacctcctgtcagatcaactgcaccatgagagtctcataggagtagaaaccctagtgtggactgcacatgcgagggaaggactgacgtcactcagtcctccttactaactttttttccctttttttccAGgacggcagcgtgcagctcgccgaccactaccagcagaacacccccatcggcgacggccccgtgctgctgcccgacaaccactacctgagctaccagtccgccctgagcaaagaccccaacgagaagcgcgatcacatggtcctgctggagttcgtgaccgccgccgggatcactctcggcatggacgagctgtacaaggggggtggaggctctgagcagaagctgatcagcgaggaggacctg
**SF2/ASF:ACT:K 6.5**	**6**	ccacagtagtttttcgtaagtataggtcctACTaagaacagaaatgaactgggctgcacagcaggaggtaagtggcaggtgagcgagcattacctggcttgagctctacctcctgtcagatcaactgcaccatgagagtctcataggagtagaaaccctagtgtggactgcacatgcgagggaaggactgacgtcactcagtcctccttactaactttttttccctttttttccAGgacggcagcgtgcagctcgccgaccactaccagcagaacacccccatcggcgacggccccgtgctgctgcccgacaaccactacctgagctaccagtccgccctgagcaaagaccccaacgagaagcgcgatcacatggtcctgctggagttcgtgaccgccgccgggatcactctcggcatggacgagctgtacaaggggggtggaggctctgagcagaagctgatcagcgaggaggacctg

Table S6: **A list and DNA sequences of all asRNAs tested in this study.**

asRNA ID	Intron	U1-U7 cassette: asRNA sequence
**Srp40**	**2**	aaactgttgctgccagtgactaacagccgcttaatttttggagcaggttttctgacttcggtcggaaaacccct
**SC35**	**2**	aacagaatggattccagagtccaggtaagactaatttttggagcaggttttctgacttcggtcggaaaacccct
**SF2/ASF**	**2**	aaccacatcctcttcctcaggattgcctttacaatttttggagcaggttttctgacttcggtcggaaaacccct
**BP1**	**2**	aaactccatcaaatcagctataaatacgaaacaatttttggagcaggttttctgacttcggtcggaaaacccct
**BP2**	**2**	aagaaacagtattagcattagtagattggaaaaatttttggagcaggttttctgacttcggtcggaaaacccct
**BP3**	**2**	aagtccaactccatcaaatcagctataaatacaatttttggagcaggttttctgacttcggtcggaaaacccct
**BP4**	**2**	aagccatgtccaactccatcaaatcagctataaatttttggagcaggttttctgacttcggtcggaaaacccct
**BP4/Srp40**	**2**	aagccatgtccaactccatcaaatcagctataactgttgctgccagtgactaacagccgcttaatttttggagcaggttttctgacttcggtcggaaaacccct
**BP4/SC35**	**2**	aagccatgtccaactccatcaaatcagctatacagaatggattccagagtccaggtaagactaatttttggagcaggttttctgacttcggtcggaaaacccct
**Srp40/SC35**	**2**	aaactgttgctgccagtgactaacagccgcttcagaatggattccagagtccaggtaagactaatttttggagcaggttttctgacttcggtcggaaaacccct
**BP4/Srp40/SC35**	**2**	aagccatgtccaactccatcaaatcagctataactgttgctgccagtgactaacagccgcttcagaatggattccagagtccaggtaagactaatttttggagcaggttttctgacttcggtcggaaaacccct
**Srp40**	**5**	aaggttgtggagagttgtaatggcataaaacaatttttggagcaggttttctgacttcggtcggaaaacccct
**SC35**	**5**	aaggcataaaacaacacagaatccactggtgaatttttggagcaggttttctgacttcggtcggaaaacccct
**SF2/ASF**	**5**	aagaaggcagtctgtcgtaatagccaagaatttttggagcaggttttctgacttcggtcggaaaacccct
**Srp55**	**5**	aaagctccttcttgatgtaataaaaggaatttttggagcaggttttctgacttcggtcggaaaacccct
**BP1**	**5**	aagcctcatcagaaatattgtgagtatacaatttttggagcaggttttctgacttcggtcggaaaacccct
**BP2**	**5**	aagggaagaagaaaaaagcctcatcagaaataaatttttggagcaggttttctgacttcggtcggaaaacccct
**BP3**	**5**	aaggtgaactgggaagaagaaaaaagccaatttttggagcaggttttctgacttcggtcggaaaacccct
**Srp40**	**6**	aacttcagcactctgcttgtggtccacagtagaatttttggagcaggttttctgacttcggtcggaaaacccct
**SC35**	**6**	aattggggtccaccactagccagtatgatgagaatttttggagcaggttttctgacttcggtcggaaaacccct
**SF2/ASF**	**6**	aaccacagtagtttttcgtaagtataggtcctaatttttggagcaggttttctgacttcggtcggaaaacccct
**BP1**	**6**	aatatatatatcttgttagtcaaggaatcttgaatttttggagcaggttttctgacttcggtcggaaaacccct
**BP2**	**6**	aaagaaagatatatatatatatcttgttagtcaatttttggagcaggttttctgacttcggtcggaaaacccct
**BP3**	**6**	aatgatgagctagaaagatatatatatatatcaatttttggagcaggttttctgacttcggtcggaaaacccct
**BP4**	**6**	aacaccactagccagtatgatgagctagaaagaatttttggagcaggttttctgacttcggtcggaaaacccct
**BP4/Srp40**	**6**	aacaccactagccagtatgatgagctagaaagcttcagcactctgcttgtggtccacagtagaatttttggagcaggttttctgacttcggtcggaaaacccct
**BP4/SF2/ASF**	**6**	aacaccactagccagtatgatgagctagaaagccacagtagtttttcgtaagtataggtcctaatttttggagcaggttttctgacttcggtcggaaaacccct
**SF2/ASF/Srp40**	**6**	aaccacagtagtttttcgtaagtataggtcctcttcagcactctgcttgtggtccacagtagaatttttggagcaggttttctgacttcggtcggaaaacccct
**BP4/SF2/ASF/Srp40**	**6**	aacaccactagccagtatgatgagctagaaagccacagtagtttttcgtaagtataggtcctcttcagcactctgcttgtggtccacagtagaatttttggagcaggttttctgacttcggtcggaaaacccct

Table S7: **Transfection mixtures used in this study.**

Main fig.	Plasmid name	Amount (ng)	Total amount	Suppl. Fig.	Plasmid name	Amount (ng)	Total amount
**Figure 1 D,E,F**	iRFP	50	**1000 ng per well in 24 well plate**	**Figure S4A,S4B**	iRFP	50	**1000 ng per well in 24 well plate**
	Target inton reporter	200			Target inton reporter	200	
	PTM	400			PTM	400	
	pcDNA3	350			pcDNA3	350	
	**Negative control (target only)**				**Negative control (target only)**		
	iRFP	50			iRFP	50	
	Target inton reporter	200			Target inton reporter	200	
	pcDNA3	750			pcDNA3	750	
	**Negative control (no target intron reporter)**				**Negative control (no target intron reporter)**		
	iRFP	50			iRFP	50	
	PTM	400			PTM	400	
	pcDNA3	550			pcDNA3	550	
**Figure 2 D,E/ Figure 3A/ Figure 4D/Figure 5A, 5B, 5D/ Figure 6 A-C**	iRFP	50	**1050 ng per well in 24 well plate**	**Figure S2C-D/Figure S7B-D/Figure S8/Figure S9/Figure S10A-E**	iRFP	50	**1050 ng per well in 24 well plate**
	Target inton reporter	200			Target inton reporter	200	
	PTM	400			PTM	400	
	asRNA	400			asRNA	400	
	pcDNA3	0			pcDNA3	0	
	**Negative control (target only)**				**Negative control (target only)**		
	iRFP	50			iRFP	50	
	Target inton reporter	200			Target inton reporter	200	
	pcDNA3	800			pcDNA3	750	
	**Negative control (no target intron reporter)**				**Negative control (no target intron reporter)**		
	iRFP	50			iRFP	50	
	PTM	400			PTM	400	
	asRNA	400			asRNA	400	
	pcDNA3	200			pcDNA4	200	
**Figure 3C, 3E, 3F,3G**	Target intron reporter	150	**1000 ng per well in 24 well plate**	**Figure S5B/Figure S7A**	Target inton reporter	150	**1000 ng per well in 24 well plate**
	PTM	300			PTM	300	
	asRNA	300			asRNA	300	
	pcDNA3	250			pcDNA3	250	
	**Negative control (target only)**				**Negative control (target only)**		
	Target intron reporter	150			Target inton reporter	150	
	pcDNA3	850			pcDNA3	850	
**Figure 5C**	pcDNA3	600	**1000 ng per well in 24 well plate**	/	/		/
	PTM	400					

**Figure 4B**	iRFP	50	**1050 ng per well in 24 well plate**
	Target inton reporter	200	
	PTM	400	
	asRNA	400	
	pcDNA3	0	
	**Negative control (target only)**		
	iRFP	50	
	Target inton reporter	200	
	pcDNA3	750	
**Figure 4E**	Target inton reporter	150	**1000 ng per well in 24 well plate**
	PTM	300	
	asRNA	300	
	pcDNA3	250	
	**Negative control (target only)**		
	Target inton reporter	150	
	pcDNA3	850	
**Figure 4F**	Target intron reporter	37,5	**198,75 ng per well in 96 well plate**
	TopFlash	18,75	
	phRL-TK	5	
	PTM	75	
	pcDNA3	62,5	
	**Negative control (without target)**		
	TopFlash	18,75	
	phRL-TK	5	
	PTM	75	
	pcDNA3	100	

**Figure S4E-F**	iRFP	50	**1050 ng per well in 24 well plate**
	Target inton reporter	200	
	PTM	400	
	asRNA	400	
	pcDNA3	0	
	iRFP	50	
	Target inton reporter	200	
	**combined PTM**	800	
	pcDNA3	0	
	**Negative control (target only)**		
	iRFP	50	
	Target inton reporter	200	
	pcDNA3	750	
	**Negative control (no target intron reporter)**		
	iRFP	50	
	PTM	400	
	asRNA	400	
	pcDNA4	200	
**Figure S6**	Target inton reporter	100	**500 ng per well in 8 well plate**
	PTM	200	
	asRNA	200	
	pcDNA3	0	
	**Negative control (target only)**		
	Target inton reporter	100	
	pcDNA3	400	

Table S8: **qPCR and semi-qPCR primer sequences.**

**qPCR and semi-quantitative PCR primers**	**Primer sequence (5´--> 3´)**	**Amplicon size (bp)**
Semi-quantitative PCR amplification of trans-spliced RNA segments	gtgagcaagggcgaggagctg (forward)<br>caggtcctcctcgctgatcagct (reverse)	759
qPCR amplification of trans-spliced RNA segments	caagatccgccacaacatcg (forward)<br>caggtcctcctcgctgatca (reverse)	265
qPCR amplification of cis-spliced RNA segment in intron 2 reporter	caagatccgccacaacatcg (forward)<br>aggtaagactgttgctgcca (reverse)	102
qPCR amplification of cis-spliced RNA segment in intron 5 reporter	caagatccgccacaacatcg (forward)<br>cttgctttcttggttgccataag (reverse)	225
qPCR amplification of cis-spliced RNA segment in intron 6 reporter	caagatccgccacaacatcg (forward)<br>tgcattccaccagcttctac (reverse)	178
qPCR amplification of housekeeping gene GAPDH as control	Gaaggtgaaggtcggagtc (forward)<br>Gaagatggtgatgggatttc (reverse)	240
Semi-quantitative PCR amplification of endogenous trans-spliced RNA segments for the intron 2	Ggtatttgaagtataccatacaac (forward)<br>Caggtcctcctcgctgatcagct (reverse)	2435
Semi-quantitative PCR amplification of endogenous trans-spliced RNA segments for the introns 5	gctgcagttatggtccatcagc (forward)<br>Caggtcctcctcgctgatcagct (reverse)	1878
Semi-quantitative PCR amplification of endogenous trans-spliced RNA segments for the introns 6	Gtggtggttaataaggctgcag (forward)<br>Caggtcctcctcgctgatcagct (reverse)	1575
